# New perspectives on picocyanobacteria and understudied cyanobacterial diversity in the Albemarle Pamlico sound system, North Carolina, USA

**DOI:** 10.3389/fmicb.2025.1539050

**Published:** 2025-05-09

**Authors:** Joel Sánchez-Gallego, Nathaniel P. Curtis, Hans W. Paerl, Ryan W. Paerl

**Affiliations:** ^1^Department of Marine, Earth, and Atmospheric Sciences, North Carolina State University, Raleigh, NC, United States; ^2^Coiba Scientific Station, City of Knowledge, Clayton, Panama; ^3^Department of Earth, Marine and Environmental Sciences, Institute of Marine Sciences, University of North Carolina at Chapel Hill, Morehead City, NC, United States

**Keywords:** cyanobacteria, picocyanobacteria, *Albemarle-Pamlico Sound System*, *core microbiome*, *flow cytometry*, cyanotoxic populations

## Abstract

Cyanobacteria are important primary producers, sources of secondary metabolites, and sentinels of environmental change in aquatic ecosystems – including large estuaries. Here, we newly investigated cyanobacterial diversity within the Albemarle Pamlico Sound System (APES) using (16S rRNA) gene amplicon sequencing analyses. Substantial cyanobacterial diversity including lineages lacking current isolates were recovered (46 genera, 17 potentially cyanotoxic), with oligohaline waters of the Albemarle Sound and its tributaries being notable regional hotspot for diversity. Salinity and temperature were influential drivers of cyanobacterial community composition. Picocyanobacteria (cells <3 µm in diameter) were abundant in amplicon sequence libraries (72% of cyanobacterial sequences) – especially populations within *Synechococcus* SubClade 5.2. Picocyanobacteria along with picoeukaryotes were large contributors to total phytoplankton biomass comprising ~47% of chlorophyll a. Further, the picocyanobacterial genera *Synechococcus*, *Cyanobium*, and *Synechocystis* (55.4%, 14.8%, and 12.9% of cyanobacterial sequences, respectively) formed a core community spanning from freshwater regions (eastern AST, D949) to polyhaline environments (NRE100 downstream stations to PS5), suggesting resilience to significant salinity fluctuations and associated environmental changes. Amplicon sequence variant (ASV) and environmental data indicate the presence of several putative ecotypes, as well as distinct abundance patterns among closely related populations, highlighting substantial fitness variability among subspecies. Notably, potentially cyanotoxic genera, *Synechocystis*, *Planktothrix*, *Plectonema*, and *Dolichospermum* were the four more abundant detected in polyhaline APES regions, far beyond conspicuous freshwater sources. These findings reveal previously unrecognized potential sources of cyanotoxics in estuarine food webs and habitats, underscoring the ecological significance of cyanobacterial community dynamics across salinity gradients.

## Introduction

Cyanobacteria occur in diverse aquatic systems and play key ecosystem roles, including contributing significantly to primary production and affecting water quality (Fogg et al., 1973; Li, 1994; Paerl et al., 2001). Substantial cyanobacterial diversity exists across aquatic ecosystems (Gaysina et al., 2019), but arguably, their diversity and functional roles are less resolved in estuaries (Fogg et al., 1973; Paerl and Dubravko, 2022). Nonetheless, estuarine cyanobacteria, especially picocyanobacteria (cyanobacteria <2–3 μm in diameter), are increasingly recognized as significant contributors (e.g., >50%) to estuarine phytoplankton biomass and primary production (Ray et al., 1989; Gaulke et al., 2010; Paerl et al., 2020; Zdun et al., 2021). While pelagic ocean and freshwater ecotypes are more well established (Ahlgren et al., 2020; Six et al., 2021; Rocap et al., 2002; Cabello-Yeves et al., 2022a; Park et al., 2024), recent evidence has pointed out estuarine picocyanobacterial ecotypes with putatively different lifestyles (Liu et al., 2014; Xia et al., 2017, 2023; Zufia et al., 2022; Aguilera et al., 2023).

Cyanobacteria are endemic to the Albemarle-Pamlico Sound System (APES), the second largest estuary in the mainland USA (Paerl et al., 2007), and are significant contributors to total chlorophyll a (Chl *a*) Cyanobacteria are significant contributors to total chlorophyll a (Chl a) and picophytoplankton (PicoP) in a sub-estuary, the Neuse River Estuary (NRE) - averaging from ~ 40% and > 70% during summer periods (Gaulke et al., 2010; Paerl et al., 2020). Picocyanobacteria, in particular, are numerically dominant in the NRE and likely abundant in other APES regions, yet their abundance and diversity have not been thoroughly investigated (Stanley and Hobble, 1981; Moorman et al., 2016; Brentjens and Bratt, 2023). A genetic assessment of broad APES cyanobacterial diversity holds potential for improving the resolution of cyanobacterial biodiversity and evolution, their roles in nutrient cycles and food webs, their population dynamics, and identification of unforeseen “lifestyles,” as observed in other aquatic systems (Sohm et al., 2016; Salazar et al., 2020; Novotny et al., 2023). In addition, environmental DNA sequence data can be used to study *in situ* populations that comprise a core community that persists across environmental gradients and are presumably resilient to environmental change, e.g., salinity changes (Hanski, 1982; Lindh et al., 2017; Stadler et al., 2023), an important characteristic considering that more extreme cycling of wet/dry periods is predicted for many estuaries (Kennish et al., 2023). We hypothesize that a diverse and environmentally resilient cyanobacterial core exists within the APES despite the narrow tolerance of temperature and salinity of some cyanobacteria (Celepli et al., 2017; Olofsson et al., 2020; Cabello-Yeves et al., 2022a). This core presumably plays a role in maintaining microbiome homeostasis, especially amid increasingly frequent disturbances commonly found in estuaries, such as storm events and droughts (Paerl et al., 2018a; Walker et al., 2022; Thompson et al., 2023; Kennish et al., 2024).

In this study, we examined the APES cyanobacterial diversity and the contributions of picocyanobacteria to biomass in understudied APES regions. We used established PCR primers to obtain 16S rRNA amplicon sequences from surface water samples where hydrological measurements, flow cytometry (FCM) phytoplankton enumeration, and size-fractionated Chl *a* measurements were carried out in parallel. Taken together, these data allowed for an analysis of trends in biomass contribution and sequence (relative) abundance alongside environmental changes.

## Materials and methods

### Samples and environmental data

**Figure 1:**
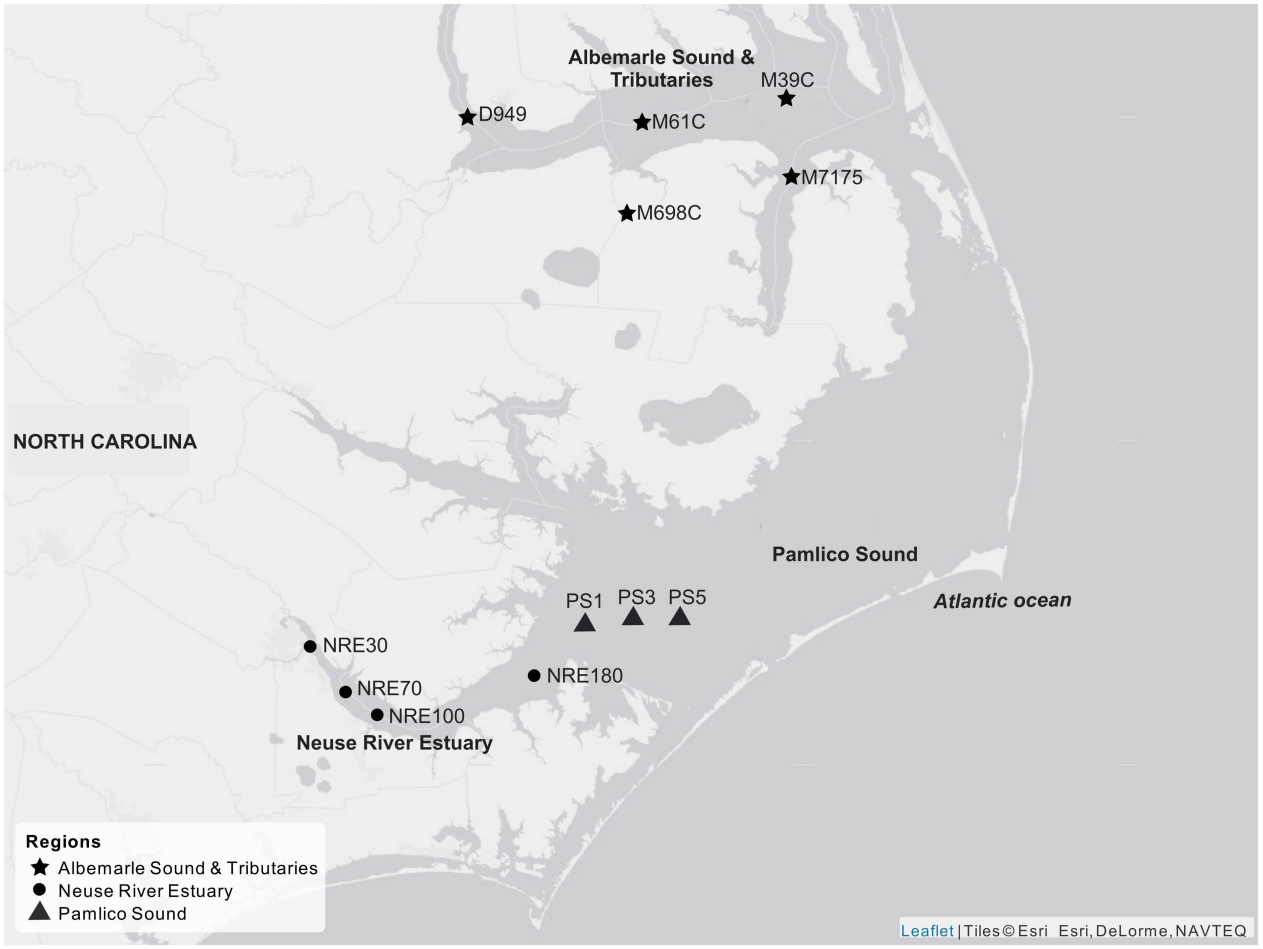
Sampled stations across the APES, including Albemarle Sound and Tributaries (AST) stations: D949, M61C, M698, M39C, and M7175; Neuse River Estuary (NRE) stations: NRE30, NRE70, NRE100, and NRE180; and Pamlico Sound (PS) stations: PS1, PS3, and PS5. Map creation credit: Esri World Gray Canvas.

Surface water was collected from NRE and Pamlico Sound (PS) stations as part of the Neuse River Estuary Modeling and Monitoring Project (ModMon) (Luettich et al., 2000; Buzzelli et al., 2001; Paerl et al., 2010), as well as from stations in Albemarle Sound and its tributaries (AST), and the North Carolina Department of Environmental Quality (NCDEQ) Ambient Monitoring Program (Figure 1). Surface water samples were kept on ice in dark coolers and transported overnight to North Carolina State University, Raleigh, NC, for processing the next day. Water samples were collected from 2017 to 2019 (Supplementary Tables S1, S2). Corresponding biological and hydrological data were provided through the ModMon and NCDEQ monitoring programs.^1^

### Flow cytometry

Samples for FCM analysis were fixed, stored, and analyzed as reported by Paerl et al. (2020) using Guava EasyCyte HT (Millipore, MA, United States) and GuavaSoft (Millipore, MA, United States). Briefly, small phytoplankton (picocyanobacteria, eukaryotic phytoplankton) were identified and enumerated based on autofluorescence from blue and red laser excitation (Liu et al., 2014; Paerl et al., 2020). The limit of quantification for FCM analysis was 1.62 × 10^1^ cells mL^−1^ based on the detection of a single cell from the average sample volume processed from winter samples. Assuming a spherical shape for all PicoP, biovolume, as well as carbon biomass units per PicoP cell, was calculated using the conversion factor of 237 fg C μm^−3^ (Worden et al., 2004).

### Chlorophyll a measurements

Total phytoplankton biomass and PicoP biomass were estimated as described by Paerl et al. (2020). Briefly, Chl *a* was extracted from biomass on Glass Fiber Filters (GF/F) filters using acetone (100%), sonication, and fluorometric analysis. Non-parametric linear regressions (Kendall–Theil) were used to evaluate the relationship between PicoP Chl *a* or percent PicoP Chl *a* and total Chl *a* via the Median-Based Linear Models (mblm v0.12.1) package (Komsta, 2019) using the R environment.

### DNA extraction and sequencing

DNA was extracted from planktonic biomass collected via vacuum filtration on Supor 0.22-μm filters (Pall; Port Washington, NY, United States) using the PowerWater Kit (Qiagen; Dusseldorf, Germany) according to the manufacturer’s instructions, with a modification of three cycles of freezing (−20°C) and thawing prior to extraction, to increase DNA yield. The V3–V4 region (~379 bp) of the 16S rRNA gene was amplified from the extracted DNA using cyanobacterial-specific primers (CYB359-F: 5’-GGGGAATYTTCCGCAATGGG-3′; CYB781-R1: 5’-GACTACTGGGGTATCTAATCCCATT-3′, and CYB781-R2: 5’-GACTACAGGGGTATCTAATCCCTTT-3′) (Nübel et al., 1997) as well as CS1 and CS2 linker sequences at the 5′ end of primers to generate sequencer-ready libraries (Naqib et al., 2018). PCR (25 μL) contained 0.2 μM of each primer and 1x KAPA HiFi HotStart Ready Mix (KAPA Biosystems, Cape Town, South Africa). Products of triplicate PCR using each sample were pooled and sequenced using Illumina Miseq V3 chemistry (2 × 250 bp; Genome Research Division, Genome Research Core, University of Illinois at Chicago).

### Sequence processing and statistical analysis

Primer sequences were removed from reads using cutadapt (v4.2) (Martin, 2011), followed by denoising and chimera removal using DADA2 (v1.26.0) (Callahan et al., 2016) (parameters: maxN = 0, maxEE = 2,5, trunclen = 260,160). Taxonomic assignment of the amplicon sequence variants (ASVs) was performed within DADA2 with the Ribosomal Database Project Bayes naïve classifier (Cole et al., 2014) against the SILVA v138 database (Quast et al., 2012) with a minimum bootstrap confidence level of 80%. After data processing, an average of 13,085 sequences per sample were retained (min: 7140, max: 190173). ASVs classified as chloroplast (40.7%) and non-cyanobacterial (33.3%) were removed. A phylogenetic tree was then constructed using the aligned sequences to verify that all were affiliated to cyanobacteria; any non-affiliated sequences were excluded from further analyses. Of the 2,441 ASVs in the library, 26% (635 ASVs) were affiliated to cyanobacteria.

All cyanobacterial ASVs (635 ASVs) identified by SILVA were reclassified using Elastic-Blast (v1.2.0) (Camacho et al., 2023). Cloud searches for each cyanobacterial ASV were conducted on the Amazon Web Server (AWS) in January 2025. Each search was performed with BLASTn against the non-redundant database. The output was saved in a tabular format (−outfmt ‘7’) using the standard scientific name as description, and the closest match with a 98% threshold of sequence identity was used for further analyses. All ASVs below the threshold were categorized as unidentified cyanobacteria. ASVs with a best BLASTn hit (with 98% of percent identity as cutoff) to a cyanobacteria reference strain were selected over environmental samples, and the final taxonomic assignation shown in figures, text, and tables was from BLASTn. The strategy of reannotating ASVs identified by DADA2/SILVA and subsequently using BLASTn (MEGABLAST) enhanced taxonomic classification at the species level of all ASVs, providing more accurate insights into cyanobacterial composition of the samples (Bars-Cortina et al., 2023). Given the well-known limitations of short-read sequencing, which can lead to misassignments when using the NCBI database for taxonomy, particularly for certain cyanobacterial taxa with percent identity values >98%, the annotation was carefully examined through additional phylogenetic analyses (Salmaso et al., 2022).

Cyanobacterial sequences were aligned using Multiple Alignment Fast Fourier Transform (MAFFT) (Katoh and Standley, 2013), and alignment gaps were removed via Block Mapping and Gathering with Entropy (BMGE) (Criscuolo and Gribaldo, 2010) using NGPhylogeny.fr (default settings) (Lemoine et al., 2019). Aligned sequences were used to construct phylogenetic trees using Seaview (Gouy et al., 2010) and the neighbor-joining method, assuming a Jukes–Cantor model for tree construction (1,000 replicates). The Jukes–Cantor model was selected due to its computational efficiency and its suitability for distance-based clustering approaches in large cyanobacterial datasets, instead of using a model selection strategy (e.g., ModelTest) that might provide a more rigorous and robust approach to phylogenetic inference. The resulting trees were edited using the interactive tree of Life (iTOL, http://itol.embl.de). References for *Synechococcales* clades were obtained from several publications (Robertson et al., 2001; Ernst, 2003; Jasser et al., 2011; Xu et al., 2015; Hunter-Cevera et al., 2016; Albrecht et al., 2017; Juteršek et al., 2017; Doré et al., 2020; Gin et al., 2021; Schallenberg et al., 2022; Suzuki et al., 2022; Aguilera et al., 2023). *Synechococcales* and non-*Synechococcales* references are included in Supplementary Tables S3, S4.

Alpha diversity metrics (observed = richness, Simpson, Shannon) were obtained using phyloseq (v1.42.0) (McMurdie and Holmes, 2013). Faith’s phylogenetic diversity indices were calculated using the picante package (v1.8.2). Sequence abundances were not rarefied to minimum sample abundance to avoid loss of information and statistical power as rarefaction can artificially reduce data complexity and introduce biases (McMurdie and Holmes, 2014). All metrics were visualized using the plot_richness function from phyloseq. Non-parametric Wilcoxon signed-rank tests (ggpubr package v0.6.0) were performed to assess significant differences in alpha diversity estimates for regions, including singletons for relevant metrics.

Abundances of ASVs were Hellinger-transformed, used to calculate the Bray–Curtis index (dissimilarities), and plotted using a non-metric multidimensional scaling (NMDS) implemented by the microviz package (v0.10.6) (Barnett et al., 2021). Statistical differences in community composition across regions and seasons were calculated with PERMANOVA using the adonis2 function with 999 permutations using the vegan package (v2.6.8). The core cyano-microbiomes of NRE-PS and AST were determined based on a minimum relative abundance of 0.1% and a prevalence threshold of 20% for NRE-PS and 40% for AST for each ASV (Hernandez-Agreda et al., 2017; Risely, 2020). Venn diagram was generated using the package microeco (v1.12.0) (Liu et al., 2021).

Relationships between environmental parameters (Z-score-transformed) and ASV abundances were examined through transformed-based redundancy analysis (tb-RDA) using vegan (Oksanen et al., 2022) and ggplot2 (v3.5.1) (Wickham et al., 2023) packages. Monte Carlo permutation was used to test the significance of each significant individual environmental variable in the structure of cyanobacterial communities across regions (NRE-PS) and seasons and across AST stations and seasons. A goodness-of-fit test (Wang et al., 2020) was carried out to identify the number of ASVs that explain a majority of variation across all samples.

Spearman’s rank correlation was performed to identify significant relationships (*p* < 0.001) between abundant ASVs of NRE-PS (top 17) and significant environmental factors determined by the RDA using the package corrplot (v0.95) in the R environment (Wei et al., 2021).

## Results

### APES cyanobacterial diversity patterns

**Figure 2:**
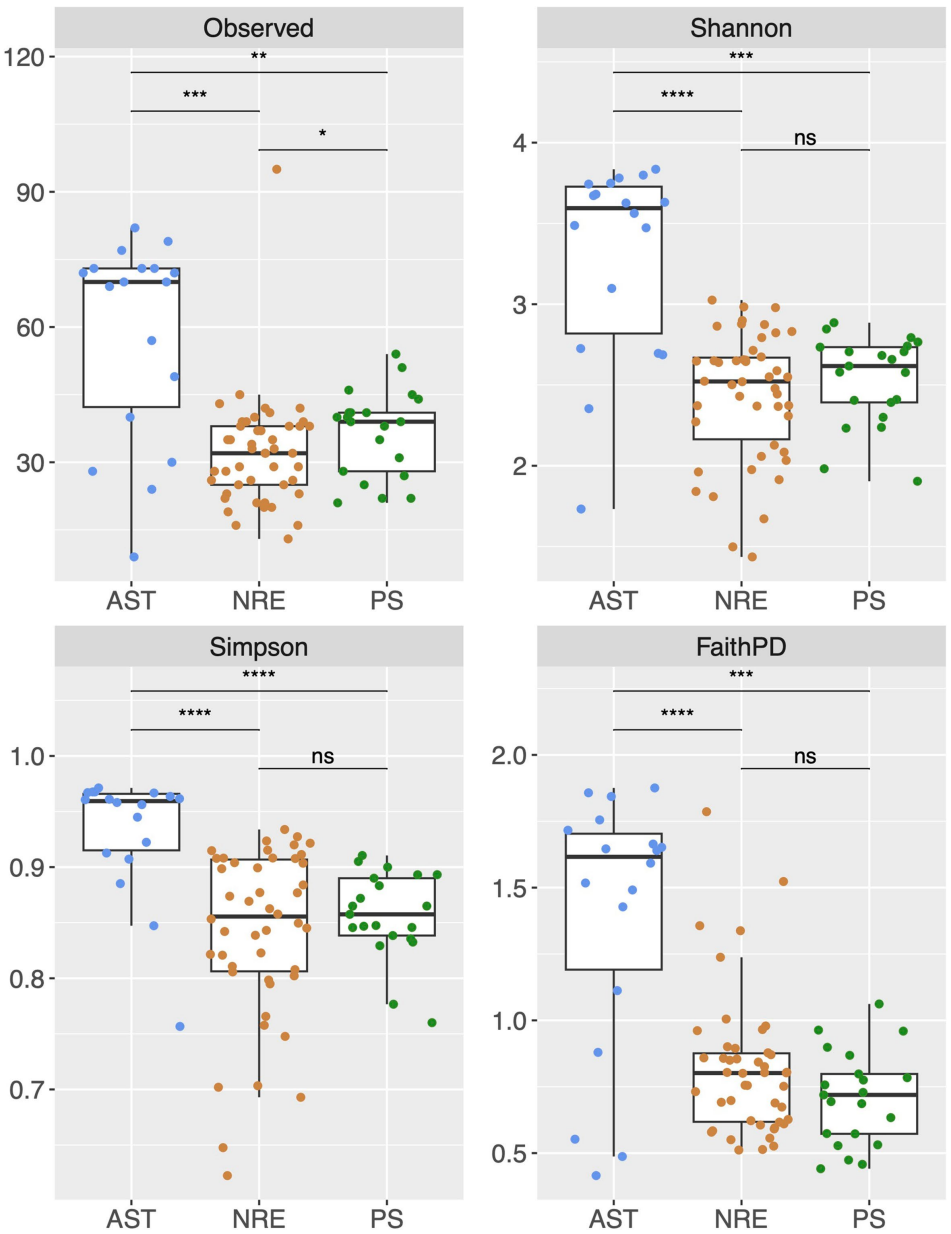
Alpha diversity indexes (at the ASV level) across regions of APES. Levels of significance determined by Wilcoxon test: 0.0001 = ****, 0.001 = ***, 0.01 = **, 0.05 = *, 1 = non-significant—ns. Observed = richness (number of different ASVs observed), Shannon H′ = − ∑i=1R'pi'∗ln'pi' where *R* is total richness, *pi* is the proportion of *R* of the *i*th species, Simpson (measure of evenness (D), D = 1- Σ*p*^2^*i*). Faith PD phylogenetic diversity = phylogenetic diversity. The data in the boxplot are depicted as first and third quartiles, and the line in the middle of each box represents the median of the relative abundance of each genus. Dots represent individual samples. “Whisker” lines above and below boxes denote the minimum and maximum value.

**Figure 3:**
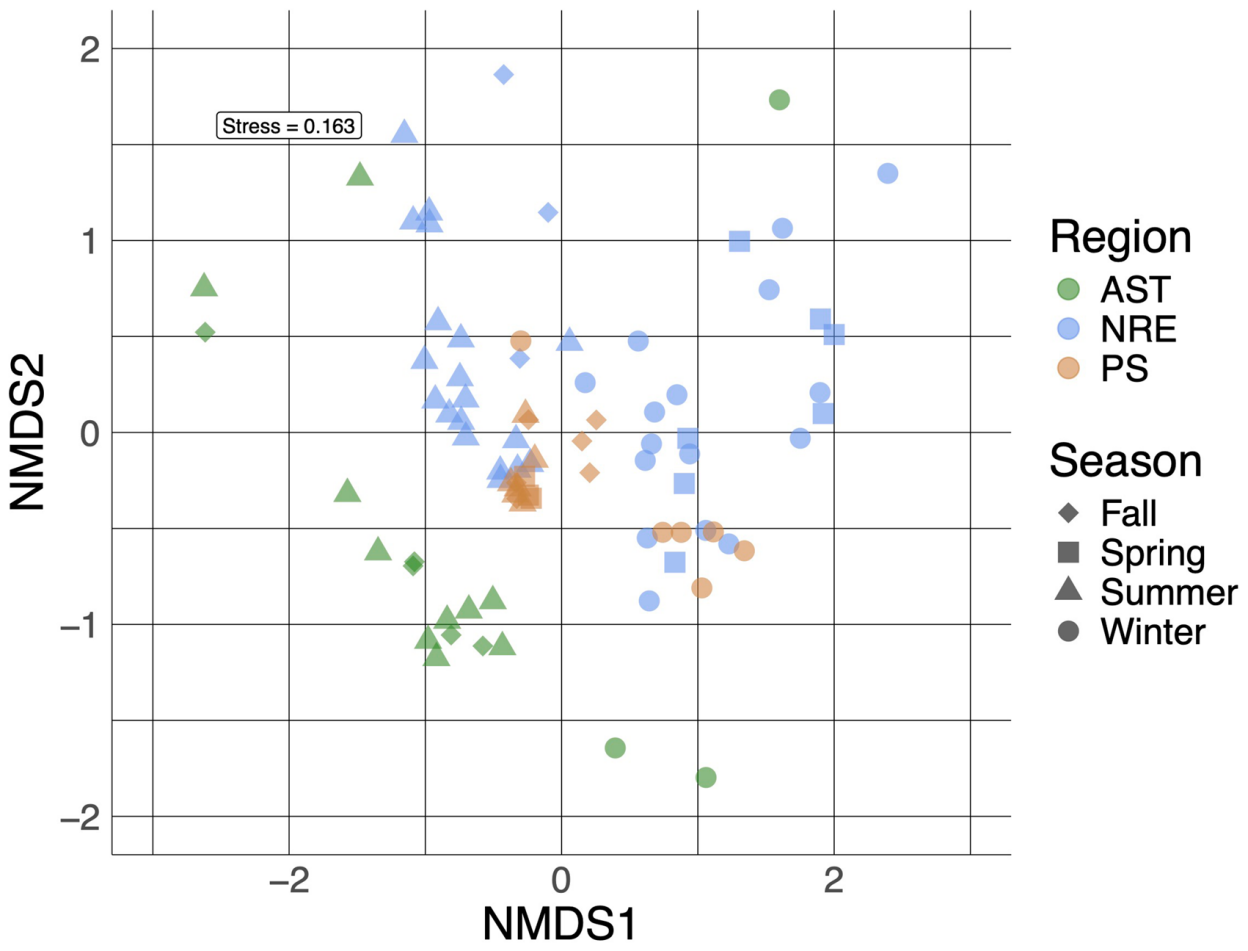
A non-metric multidimensional scaling (NMDS) plot of APES cyanobacterial communities (at the ASV level) labeled based on region and season.

Multiple alpha diversity analyses using the 635 recovered cyanobacterial ASVs revealed higher cyanobacterial diversity in AST compared to NRE-PS samples (Shannon diversity index *H′* = 3.59, 2.52, and 2.62, respectively) (Figure 2). Along the NRE-PS freshwater to polyhaline continuum, alpha diversity decreased from the upper estuary (NRE30) to middle estuary (NRE100), but it showed a slight increase at lower NRE (NRE180) and PS stations (Supplementary Figure S1). Diversity was lowest at riverine AST stations (Chowan River and Scuppernong River) and higher downriver at Albemarle Sound and Alligator River stations (Supplementary Figure S1). Cyanobacterial community composition (beta diversity) across samples was separated by season (*p* = 0.001, R^2^ = 0.20) and region (*p* = 0.001, R^2^ = 0.19) with a clear distinction of cold (winter and early spring) and warm (summer and fall) weather communities. NRE-PS communities were also distinct from AST communities (Figure 3). Of the environmental variables measured, temperature and salinity were the most significant contributors to variations in cyanobacterial diversity (combined 34.3% in NRE-PS and 14.5% in AST) (Table 1). Accordingly, most variations in APES cyanobacterial composition were not explained by commonly measured water quality parameters (~ 66–86% for NRE-PS and AST, respectively), which is in line with the results from other estuarine/marine studies (Bertos-Fortis et al., 2016; Affe et al., 2018; Ahlgren et al., 2019).

**Table 1:** Results of a Monte Carlo permutation by environmental variable and cyanobacterial communities of NRE-PS and AST.

Region	Environmental variable	Variance explained (%)	Pseudo-F	*P*-value
NRE-PS	Temperature	13.8	28.7	0.001
NRE-PS	Salinity	7.8	16.4	0.001
NRE-PS	Dissolved oxygen	1.41	2.94	0.001
NRE-PS	Turbidity	2.6	5.47	0.001
NRE-PS	Particulate organic carbon	1.4	3.12	0.003
NRE-PS	Dissolved organic carbon	1.3	2.76	0.008
NRE-PS	Dissolved inorganic carbon	1.6	3.54	0.007
NRE-PS	NO3/NO2	1.6	3.37	0.004
NRE-PS	PO4	1.1	2.31	0.027
NRE-PS	SiO2	1.3	2.82	0.009
AST	Salinity	7.0	2.62	0.018
AST	Temperature	7.4	2.78	0.015

The analysis is based on redundancy analysis, where each variable’s explanatory power is measured in terms of variance explained, pseudo-*F* values, and statistical significance (*p*-value).

### Identified cyanobacterial taxa

**Figure 4:**
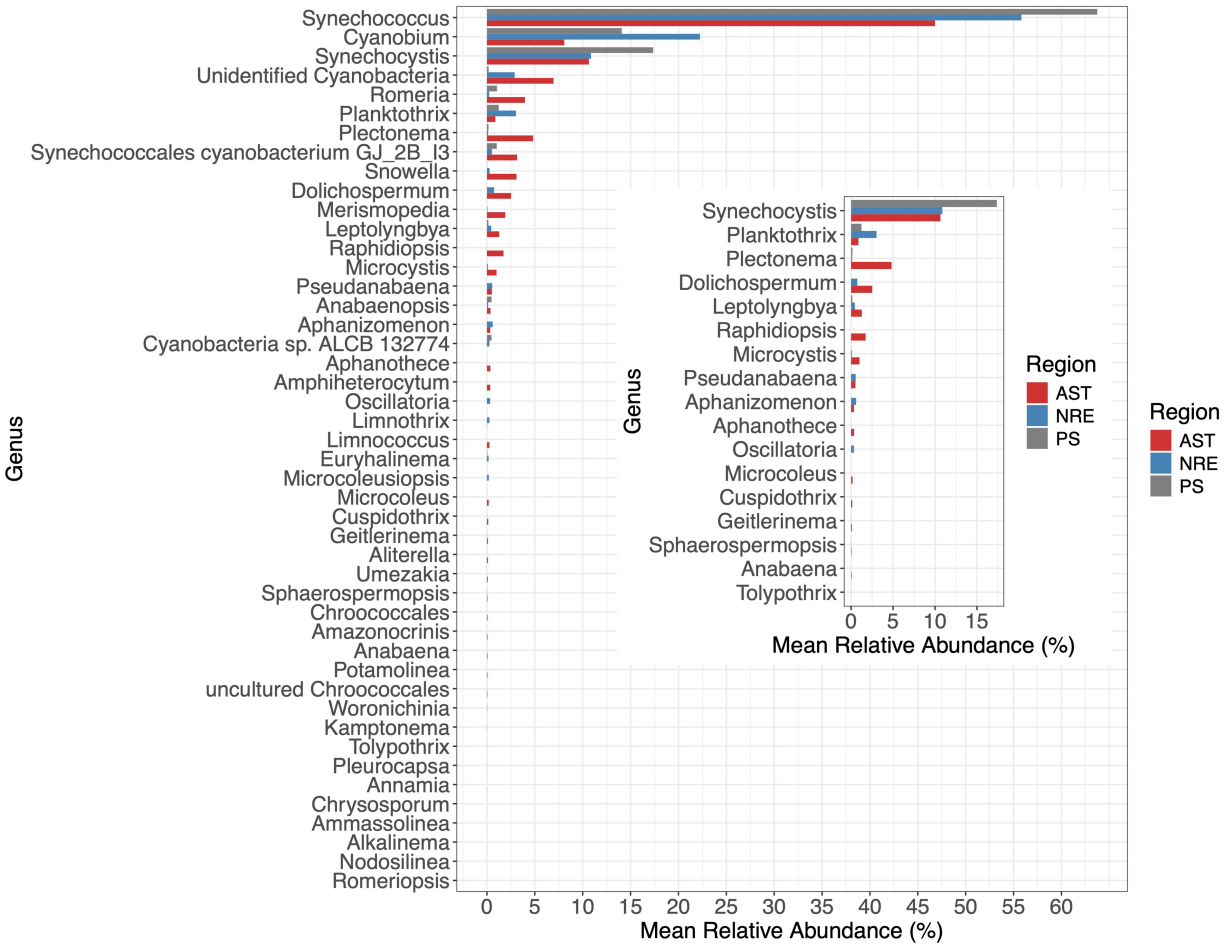
Mean relative abundance of 46 cyanobacterial genera in each region (Inset). Mean relative abundance of 17 potential cyanotoxic-producing genera in each region. The inset plot shows the mean relative abundance of potential toxin-producing genera (based on Jones et al., 2021).

**Figure 5:**
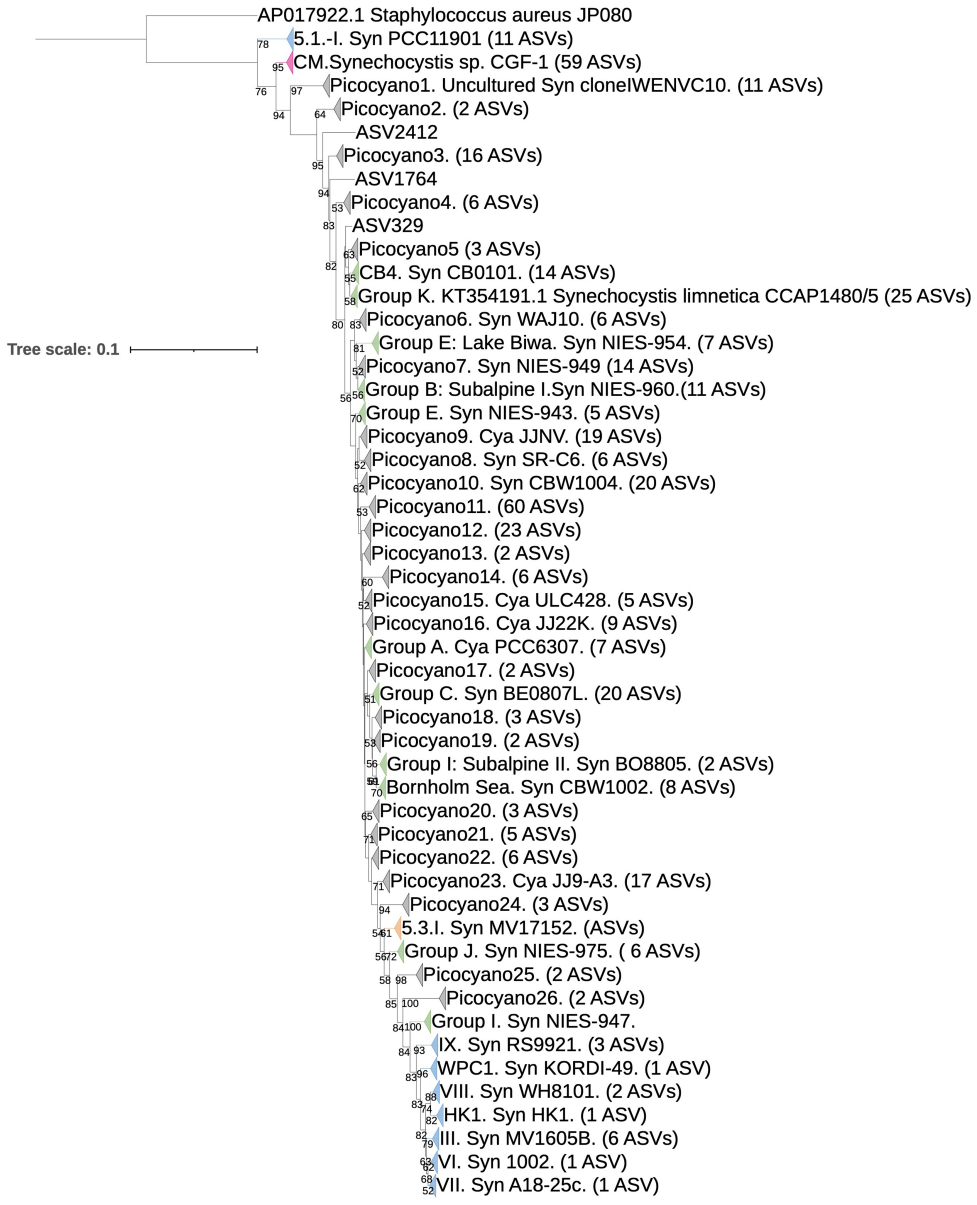
*Synechococcales* phylogenetic tree constructed from cyanobacteria-specific 16S RNA gene (V3-V4 region) sequences, including reference (*n* = 111) and 454 APES ASV sequences. Clade assignments for sequences were made by identifying the closest known group representative(s) or designated as Picocyano 1–26 if APES sequences did not cluster with strain(s) known to be affiliated with an established clade. The phylogenetic tree was rooted using the outgroup, *Staphylococcus aureus* JP080. Groups of *Synechococcu*s subclusters (SC) are shown in green (SC 5.2), blue (SC 5.1), orange (SC 5.3), pink (representing *Coelosphaeriaceae* and *Merismopediaceae: Synechocystis*, *Merismopedia*, *Snowella*, *Coleomoron*, *Woronichinia*), and grey (potential novel clades, “Picocyano”). Numbers in the tree represent the bootstrap value (bootstrap values below 50% are not shown at the nodes).

Multiple cyanobacterial groups were identified in APES sequence libraries, including unicellular and colonial forms, diazotrophs, potential cyanotoxic producers, and other poorly resolved populations (unidentified cyanobacteria, 3% of cyanobacterial sequences). In total, 46 genera were identified, and sequences belonging to the picocyanobacterial genera *Synechococcus*, *Cyanobium*, and *Synechocystis* were dominant across APES samples (55.8, 17.2, and 12.4%, respectively) (Figure 4). Notably, *Synechocystis limnetica* CCAP1480/5 and 17 related APES ASVs (e.g., ASVs 3, 8, and others; Figure 5) were classified by dada2 and BLAST as belonging to *Synechococcus* and *Cyanobiu*m (Group K) rather than to *Synechocystis* species. This clustering is consistent with recent phylogenetic analyses that suggest a close relationship between these taxa (Juteršek et al., 2017). Multiple picocyanobacterial lineages were obtained and were most phylogenetically affiliated with *Synechococcus* subcluster (SC) 5.2 (Figure 5), which was recently named the *Cyanobium* SC (Doré et al., 2020). SC 5.2 sequences clustered into 36 clades, with 26 lacking a known representative isolate or metagenome-assembled genome (MAG) and the remaining being affiliated with previously described clades, e.g., Group I, Subalpine II, and Group C (Robertson et al., 2001; Cai et al., 2010; Suzuki et al., 2022) (Figure 5). A single ASV clustered within SC 5.3, while eight ASVs were affiliated with marine SC5.1 and previously reported clades within this marine SC (SC5.1) (WPC1, HK1, I, III, IX, VI, VII, and VIII) (Figure 5).

**Figure 6:**
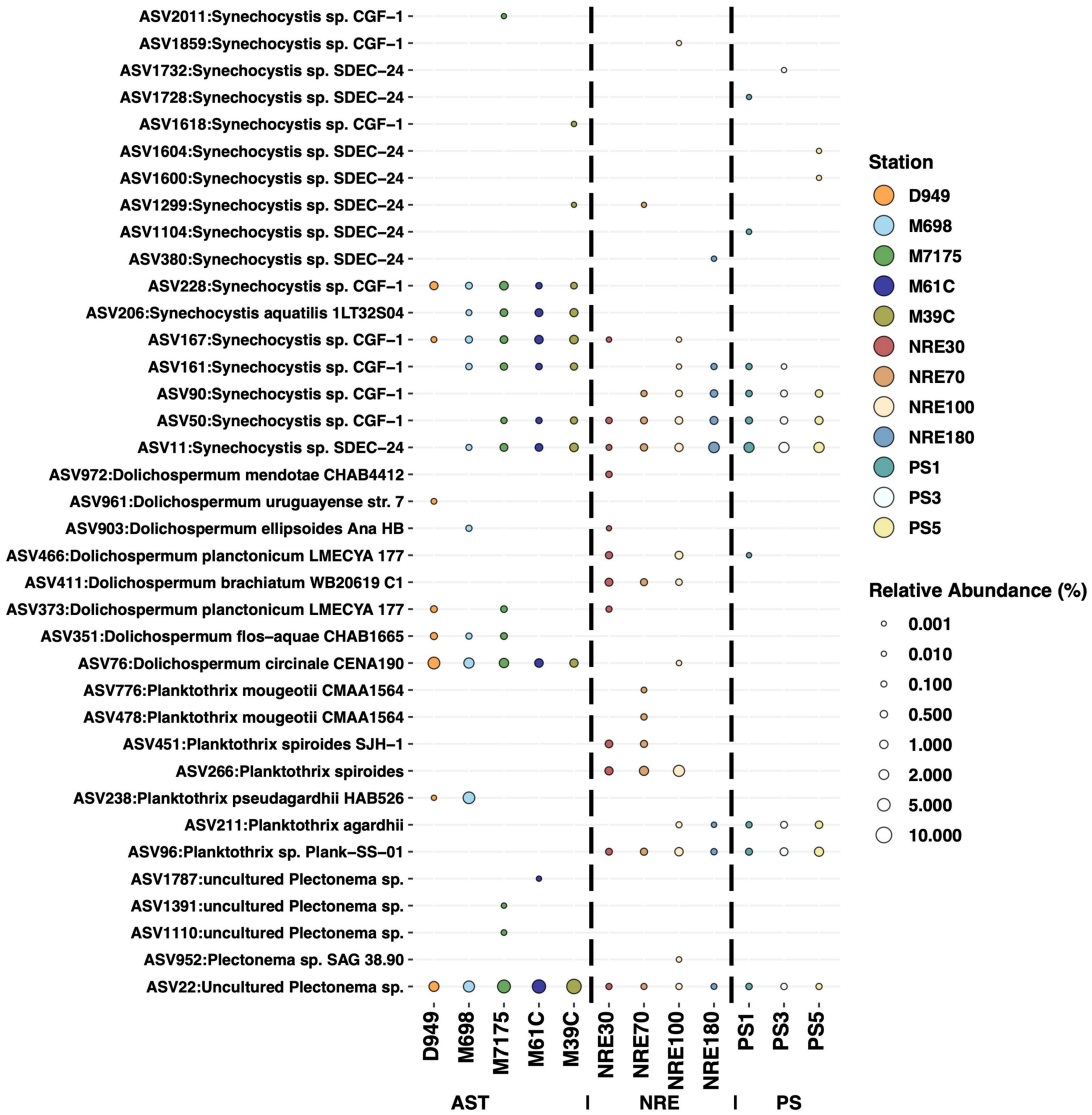
Relative abundances for 37 populations (ASVs) belonging to the four most abundant potentially cyanotoxics genera (*Synechocystis*, *Dolichospermum*, *Planktothrix*, and *Plectonema*). Each bubble represents the mean relative abundance of each ASV at different stations. All ASVs identified as *S. limnetica* CCAP1480/5 were excluded from this analysis and plot because they are more phylogenetically related to *Synechococcus/Cyanobium* (uncommon toxin producers) and therefore might not be considered cyanotoxic producers.

Notable non-picocyanobacterial clades and taxa (Supplementary Figure S2) were also identified. Among these were *Coelosphaeriaceae* and *Merismopediaceae* affiliates (referred to as the CM clade here), specifically *Coelomoron*, *Woronichinia*, *Snowella*, and *Merismopedia* populations, which are primarily considered colonial cyanobacteria. A total of 17 potentially cyanotoxic genera were detected, namely *Anabaena*, *Aphanizomenon*, *Aphanothece*, *Cuspidothrix*, *Dolichospermum*, *Geitlerinema*, *Leptolyngbya*, *Microcoleus*, *Microcystis*, *Oscillatoria*, *Planktothrix*, *Plectonema*, *Pseudanabaena*, *Raphidiopsis*, *Sphaerospermopsis*, *Synechocystis*, and *Tolypothrix* (Figure 4). Some of these genera notably occurred in higher-salinity NRE-PS waters and at times reached relatively high abundances, such as *Synechocystis* (17.3% in PS), *Planktothrix* (3.02% in NRE), *Dolichospermum* (0.74% in NRE), *Oscillatoria* (0.33% in NRE), and *Aphanizomenon* (0.59% in NRE) (Figure 4). In particular, the four most abundant and cosmopolitan of these genera (*Synechocystis*, *Planktothrix*, *Plectonema*, and *Dolichospermum*) did include individual ASVs of varying abundance across the APES (Figure 6). However, *Dolichospermum* and *Planktothrix* were more common in NRE-PS stations, whereas *Synechocystis* ASVs were the most prevalent in AST stations; in addition, *Plectonema* ASVs appeared at low abundance in nearly all monitoring stations (Figure 6).

### Picocyanobacterial abundance and contribution to Chl *a* and POC in the APES

While picocyanobacteria are recognized as abundant and significant contributors to primary production and phytoplankton biomass in the NRE (Gaulke et al., 2010; Paerl et al., 2020), they have not been studied in larger PS and AST components of the APES. Phycocyanin-rich and phycoerythrin-rich *Synechococcus*-like cells (PC-Syn and PE-Syn, respectively) and picoeukaryotic algae were present in the PS and AST samples (Supplementary Figures S3, S4). PC-Syn was the most abundant, generally matching the dominance of SC5.2 *Synechococcus* in recovered APES cyanobacterial sequences (Figure 5). PC-Syn cells reached upwards of ~7 × 10^5^ cells/mL in the summer/fall PS and AST samples, whereas PE-Syn abundance peaked at ~5 × 10^4^ cells/mL in the PS during the summer (2019) and in the AST during the winter (2018) (Table 2). Overall, PicoP biomass accounted for a notable percentage (on average 2.71%) of total POC across PS samples, with higher contributions during summer (~5%) and 3 weeks after the landfall of Hurricane Florence (6.15%, PS1, Oct. 4, 2018). Similarly, an increased PicoP contribution to POC was observed post-Florence in the NRE (Paerl et al., 2020), which signals broad PicoP stimulation in the NRE-PS after the extreme storm.

**Table 2:** PicoP average cell abundances from PS and AST stations.

Region	Station	PE-Syn (cells/mL)	PC-Syn (cells/mL)	PEuk (cells/mL)
PS	PS1	6.86E+03 ± 1.04E+04	2.30E+05 ± 1.74E+05	2.30E+05 ± 1.87E+04
PS	PS3	1.19E+04 ± 1.72E+04	2.54E+05 ± 2.69E+05	2.54E+05 ± 1.79E+04
PS	PS5	9.47E+03 ± 9.75E+03	2.71E+05 ± 1.87E+05	2.71E+05 ± 1.22E+04
AST	D949	1.24E+03 ± 1.41E+03	4.43E+04 ± 5.44E+04	6.22E+03 ± 6.10E+03
AST	M61C	1.32E+03 ± 1.23E+03	3.25E+05 ± 1.67E+05	3.83E+03 ± 1.91E+03
AST	M698	1.31E+04 ± 1.46E+04	7.98E+04 ± 1.47E+05	1.90E+03 ± 2.13E+03
AST	M39C	1.88E+03 ± 9.39E+02	3.22E+05 ± 2.39E+05	3.72E+03 ± 1.70E+03
AST	M7175	5.24E+02 ± 1.75E+02	3.74E+05 ± 2.26E+05	2.22E+03 ± 1.08E+03

Average cell abundance ± standard deviation is shown. PE-Syn = Phycoerythrin *Synechococcus*; PC-Syn = Phycocyanin *Synechococcus*; PEuk = PicoEukaryotes.

**Figure 7:**
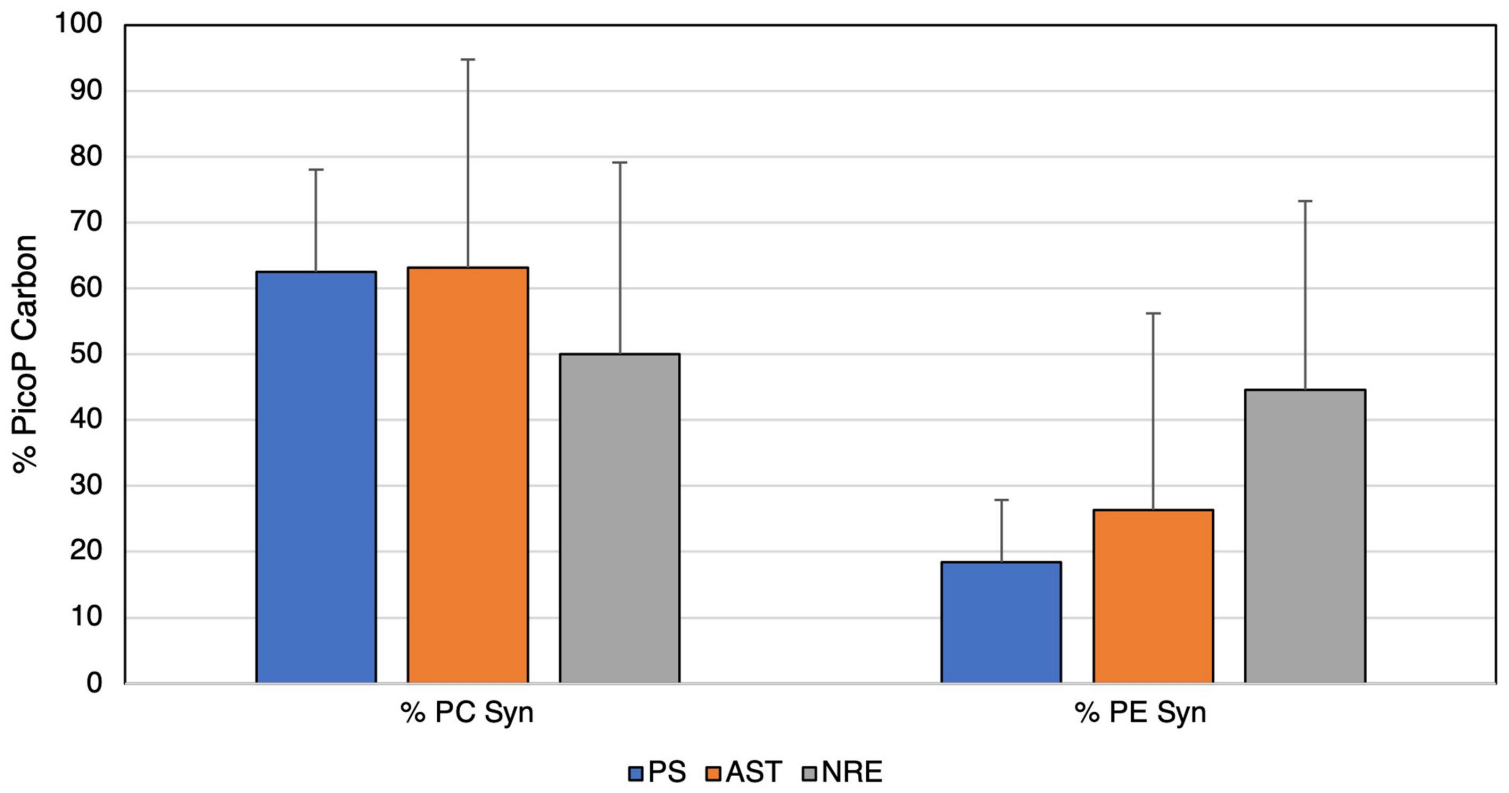
Contributions of picocyanobacteria to total PicoP biomass (carbon units) in each region of the APES.

Regarding biomass, PC-Syn cells were major contributors (up to 63%) to PicoP biomass across APES samples (Figure 7). Although PE-Syn contributed less to PicoP biomass than PC-Syn across all regions, its highest relative contribution (45%) was observed in the NRE (Figure 7). PicoP contributions to total phytoplankton biomass (extracted Chl *a*) were on average 47% (6 to 86%) in the PS and 47% (12 to 80%) in the AST (Supplementary Figures S5, S7A). In AST samples, PicoP Chl *a* concentration followed changes in total Chl *a* concentration, showing a positive linear relationship, which suggests that small and large phytoplankton respond similarly to environmental change (e.g., temperature and dissolved nutrients). Conversely, this relationship was weaker and more variable in PS samples, suggesting that PicoP and total phytoplankton biomass are influenced by distinct factors (Supplementary Figures S6, S7B).

### Select taxa (ASV) abundance patterns

**Figure 8:**
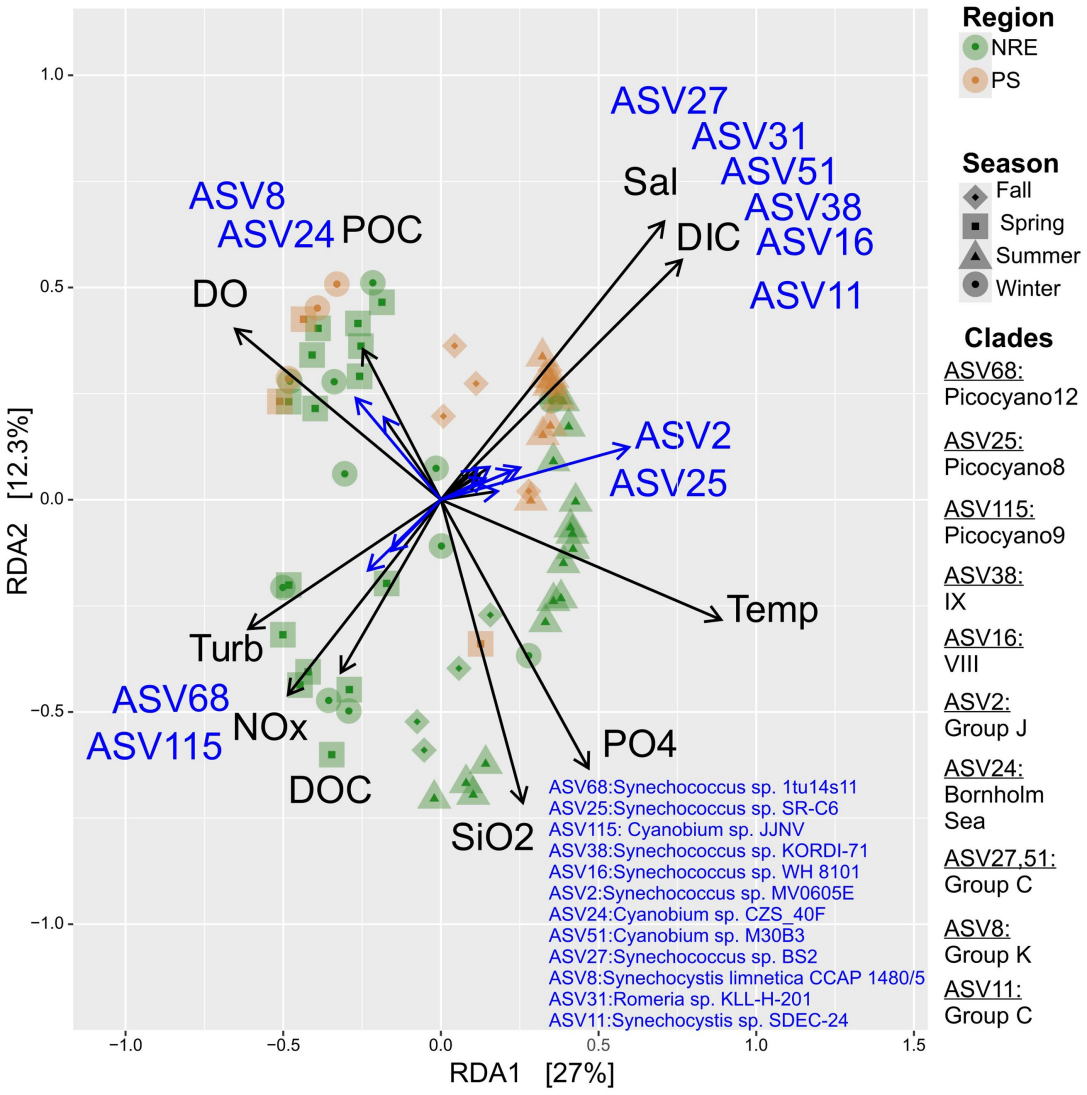
Redundancy analysis (RDA) ordination plot for NRE-PS cyanobacterial community composition in relation to environmental parameters. Only significant explanatory environmental factors (*p* ≤ 0.001) (black arrows) and ASVs (grey arrows) with goodness of fit ≥ 0.74 (grey) are included in the figure. The ASVs with goodness of fit at least 0.72 in the ordination plane formed by axes 1 and 2 are shown in the RDA plot.

Redundancy analysis, including ASV relative abundance and environmental parameters, revealed several potential ecotypes in the NRE-PS (AST data were more limited, yielding highly similar ordination patterns) (Figure 8, Supplementary Figure S8). Putative warm-temperature estuarine ecotypes (i.e., those showing positive relationships between relative abundance and temperature) were phylogenetically affiliated with (SC 5.2) clades C, J, Picocyano8, and CM (ASV27, ASV51, ASV2, ASV25, ASV11) (Figure 8). SC 5.1 ASVs were affiliated with clades IX (ASV38, *Synechococcus* sp. KORDI-71 rel.) and VIII (ASV16, *Synechococcus* sp. WH 8101 rel.), and they also showed positive relationships with temperature and salinity (Figure 8, Supplementary Figure S9). ASVs belonging to K (ASV8, *Synechocystis limnetica* CCAP 1480/5 rel.) and Bornholm Sea (ASV24, *Cyanobium* sp. CZS_40F rel.) were less abundant when the temperature was higher, suggesting they are temperate/cold ecotypes (Figure 8, Supplementary Figure S9). Finally, select ASVs (ASV 68 and 115, *Synechococcus* sp. 1tu14s11 and *Cyanobium* sp. JJNV rels.) increased with elevated levels of dissolved NOx (nitrate plus nitrite), turbidity, and dissolved organic carbon, which suggests that they may be eutrophic ecotypes (Figure 8, Supplementary Figure S9).

**Figure 9:**
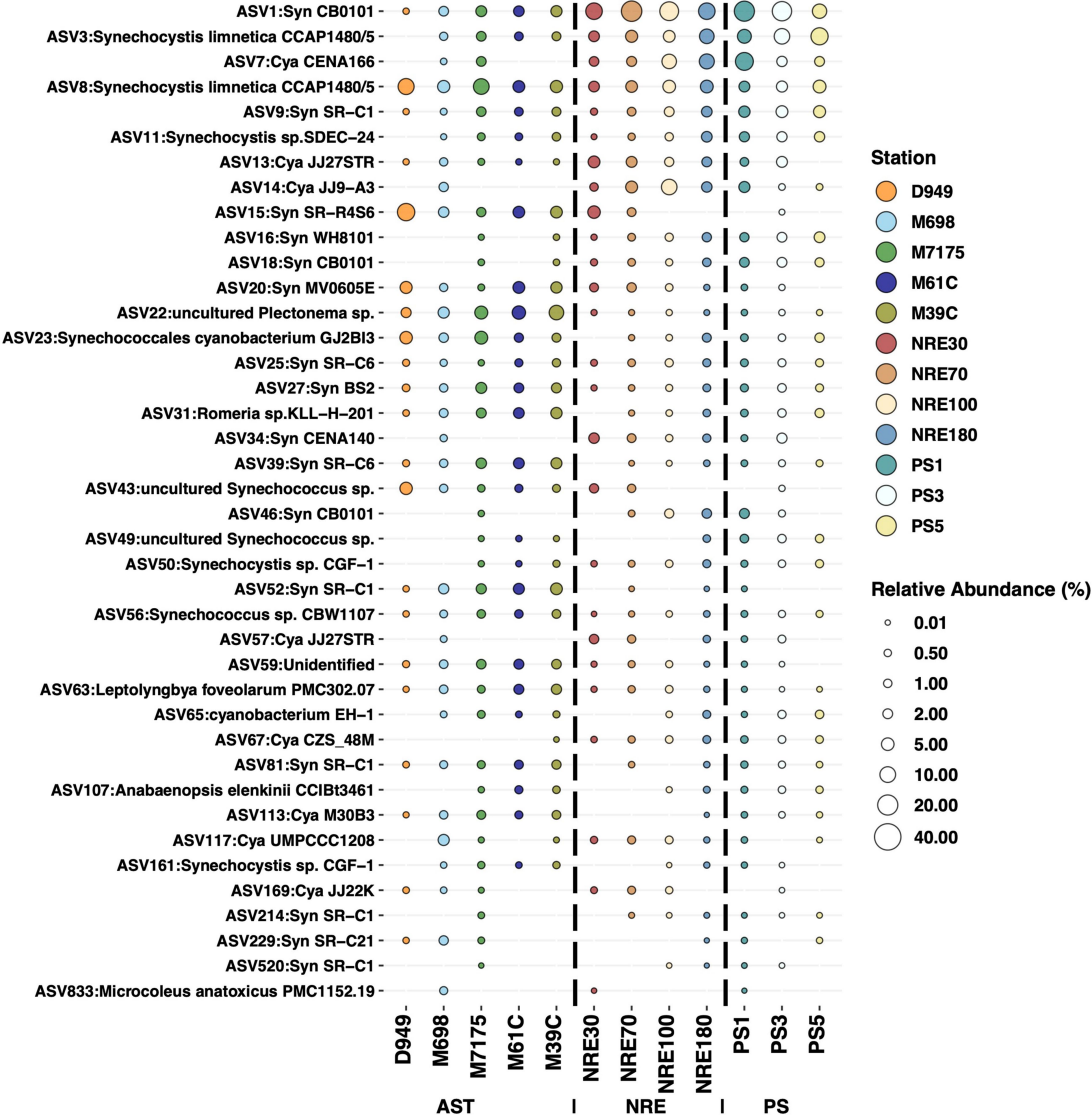
Relative abundances of the 40 shared core APES ASVs across the three regions. Samples were grouped by stations, and each bubble represents the mean of each ASV by station. ASVs were ordered based on prevalence, with the most prevalent at the top and the least prevalent at the bottom.

The most abundant cyanobacterial population based on mean relative abundance across all cyanobacterial sequence libraries (ASV1) was taxonomically affiliated with *Synechococcus* sp. CB0101 (12.4%, Supplementary Table S5), a PC-rich Chesapeake Bay isolate and representative of SC5.2 clade CB4 (Figure 9, Supplementary Figure S10) (Fucich et al., 2019). ASV1 was ubiquitous in the APES but peaked in abundance at middle estuary station NRE70 (20.8%; Figure 9, Supplementary Figure S10). Other *Synechococcus* SC5.2-affiliated ASVs were also cosmopolitan in the APES but were phylogenetically affiliated with novel clades Picocyano 8 and 12 (ASV25 and ASV9, respectively) (Supplementary Figure S10). Regionally abundant ASVs were also evident in the APES. For example, ASVs belonging to multiple *Synechococcales* clades occurred primarily in AST samples, e.g., J (ASV20), C (ASV27), A (ASV52), Picocyano 8 (ASV39), Picocyano 7 (ASV15, ASV43), and CM (ASV164) (Supplementary Figure S11). ASVs belonging to clades J (ASV2), III (ASV12), and VIII (ASV16) occurred primarily in NRE-PS samples (Supplementary Figure S12). Overall, regional and cosmopolitan picocyanobacterial populations were present in our dataset.

Furthermore, we found evidence of a shared core of cyanobacterial populations across the APES (Figure 9; Supplementary Figure S13) and several abundant populations (ASVs), e.g., ASV22 (uncultured *Plectonema* sp.) and ASV43 (uncultured *Synechococcus* sp.), that are not well represented by public 16S rRNA gene sequences from isolates or genomes (Figure 9). Regarding the shared cyanobacterial core, 40 ASVs comprised the APES cyanobacterial shared core (Figure 9, Supplementary Figure S13), and most were picocyanobacteria, e.g., *Synechococcus* (*n* = 19), *Cyanobium* (*n* = 8), and *Synechocystis* (*n* = 5). The remaining core members were either filamentous taxa, namely *Anabaenopsis* (*n* = 1), *Leptolyngbya* (*n* = 1), *Microcoleus* (*n* = 1), *Plectonema* (*n* = 1), and *Romeria* (*n* = 1), or unclassifiable at the genus level (*n* = 3). These core members are presumably highly adaptive and/or resilient to environmental changes given their breadth of occurrence across sampling stations and conditions (Supplementary Table S5).

## Discussion

### Collectively and locally rich cyanobacterial diversity within the APES

The sequence data obtained in this study highlight the APES as a system rich in cyanobacterial diversity, building on prior microscopic data and recently obtained sequence data from the APES (Piehler et al., 2002; Cira et al., 2016; Moorman et al., 2016; Plaas et al., 2022). In particular, our results open up new research avenues and further our understanding: specifically, the AST as a regional hotspot for cyanobacterial diversity; how multiple picocyanobacterial lineages coexist, especially abundant representatives of clades CB4 and Bornholm Sea resolving the ecologies of multiple genera not previously described in the APES, including picocyanobacteria and potential cyanotoxic populations (*Planktothrix*, *Plectonema*, etc.), which persist into polyhaline waters (downriver NRE-PS stations); and also relatively prevalent yet poorly classified taxa not represented by currently available isolates and genome. Whereas amplicon-based diversity studies have their limitations and do not provide metabolic insights (Ju and Zhang, 2015), metagenomic sequencing may miss portions of *in situ* cyanobacterial community diversity (Rodríguez-Gijón et al., 2023). Thus, there is significant value in the (16S rRNA sequence) diversity data obtained in our study. Moreover, a number of populations identified here serve as logical targets for future studies aiming to resolve their lifestyles and metabolic potential through *in situ* sampling and experimentation (Schallenberg et al., 2022), representative isolate acquisition and physiological laboratory studies (Xu et al., 2015; Xia et al., 2020; Schallenberg et al., 2022; Kling et al., 2023), or (meta)genomic approaches (Kearney et al., 2021; Xia et al., 2023).

Notably, the understudied AST portion of the APES is a regional hotspot for cyanobacterial diversity, making it attractive for natural product bioprospecting (Demay et al., 2019), as well as further exploration into cyanobacterial evolution and ecology (Celepli et al., 2017; Affe et al., 2018). The lowest diversity among AST samples was observed at Chowan and Scuppernong River stations (D949, M698C), which seems to reflect stronger selection against multiple cyanobacteria in these upriver stations, possibly due to increased levels of heavy metals and/or inhibitory dissolved organic matter (Preston and Shackelford, 2002; Sha et al., 2025). Overall, the high cyanobacterial diversity in AST waters, along with the increased concern in water and air quality in the region (Plaas et al., 2022; Pierce et al., 2024), generally calls for greater attention to cyanobacteria within these freshwater regions of the APES.

### APES picocyanobacteria: key contributors to diversity and biomass

Our findings show that picocyanobacteria (also PicoP) are important primary producers (2.71% of POC; upwards of 47% of Chl *a*) in the APES, which expands our prior knowledge of their importance only within the NRE (Gaulke et al., 2010; Paerl et al., 2020). The dominance of PC-Syn is in line with recent findings from NRE surface waters (Paerl et al., 2020) and calls for a greater understanding of PC-Syn ecology alongside other recent coastal and estuarine studies (Rajaneesh and Mitbavkar, 2013; Li et al., 2019; Alegria Zufia et al., 2021).

The dominance of picocyanobacterial phylogenetic clades CB4, Bornholm Sea, J, and K (*Cyanobium* clades SC 5.2, *sensu stricto*) (Doré et al., 2020) in the APES emphasizes the success of these lineages in temperate estuaries and coastal waters worldwide (Chen et al., 2006; Xu et al., 2015; Hunter-Cevera et al., 2016; Hunter-Cevera et al., 2021). Their importance in the study region and globally supports future work to understand the physiological reasons for their success, e.g., mechanisms of cell persistence (Fucich and Chen, 2020). Novel picocyanobacterial clades described in this study and in recent studies (using different PCR primers) (Zufia et al., 2022; Aguilera et al., 2023) spotlight the high picocyanobacterial microdiversity and potential for varied ecologies within SC 5.2.

The large differences we observed in the relative abundance of highly related cyanobacterial populations (ASVs), e.g., *Synechococcus* sp. CB0101 (Clade CB4) and *Synechocystis limnetica* sp. CCAP1480/5 (Clade K) ASVs, point to fitness variations among closely related clade members and emphasize that clade-level assessments of biogeography and ecology, based on relationships between *in situ* abundance and environmental variables, are likely applicable only to the most abundant representatives (Liu et al., 2014; Xu et al., 2015; Hunter-Cevera et al., 2016). Potentially, comparative genomics of representative isolate genomes or *in situ* genomes, e.g., MAGs (Xia et al., 2023), may reveal gene repertoire variations among these phylogenetically closely related taxa and help explain the large differences in their *in situ* abundance. Estuarine picocyanobacterial ecotypes are recognized based on temperature, salinity, pigmentation/photosynthetic capacity, and metal tolerance (Larsson et al., 2014; Marsan et al., 2017; Grébert et al., 2018), and similarly, the APES appears to contain different ecotypes (e.g., warm, temperate/cold; Figure 8). Measurements beyond those made by common water quality monitoring efforts may help explain variations in closely related taxa, e.g., susceptibility to viral infections (Ahlgren et al., 2019; Dart et al., 2023).

Only recently were *Synechocystis* and colonial genera *Romeria, Snowella,* and *Merismopedia* reported from a component of the APES (Chowan River) (Plaas et al., 2022); in the present study, we note that these colonial genera can be abundant (up to 1.22, 0.81, and 0.42% of cyanobacterial sequences, respectively) and deserve future study. Because these genera are often larger than *Synechococcus* and *Cyanobium* spp. and form aggregates or filaments (Callieri et al., 2012), their impact on elemental cycling (especially, particulate carbon), food webs, and water quality is potentially distinct from those of unicellular picocyanobacteria. Their larger cell size is particularly significant since larger cells tend to possess larger genomes with more biosynthetic gene clusters, for instance those coding for secondary metabolite (e.g., cyanotoxic) production (Medema et al., 2021). Furthermore, it is presumed that some *Synechocystis* spp. produce cyanotoxics (Banack et al., 2012; Cervantes Cianca et al., 2012; Vareli et al., 2013), and colonial cyanobacteria can carry out unique biochemical transformations influencing ecosystem functionality and productivity, e.g., N_2_ fixation (Bergman et al., 2006; Zehr and Paerl, 2008; Marcarelli et al., 2022).

### Unforeseen cyanotoxic populations in the APES

We detected several cyanobacterial genera with toxin-producing potential not reported previously in the APES (Plaas et al., 2022; Brentjens and Bratt, 2023) but are of concern in freshwater to marine systems (Chorus et al., 2021; Jones et al., 2021). They are *Aphanothece*, *Cuspidothrix*, *Geitlerinema*, *Microcoleus*, *Oscillatoria*, *Planktothrix*, *Plectonema, Sphaerospermopsis*, and *Synechocystis* and are logical targets for future APES water quality monitoring and research. The data also bring to light questions of whether some populations are viable and persist in the face of varied conditions along the fresh to polyhaline continuum, especially the more cosmopolitan *Synechocystis*, *Planktothrix*, *Plectonema,* and *Dolichospermum* ASVs, or whether these cells are non-viable and are transported downriver (Mohamed, 2013; Rastogi et al., 2015; Buratti et al., 2017; Paerl et al., 2018a). If they are highly adaptive, they must be physiologically unique because most of the potentially cyanotoxic genera are significantly less abundant in more saline NRE-PS surface waters. This observation is in line with prior reports of few cyanotoxic populations in polyhaline and marine waters (Tonk et al., 2007; Rosen et al., 2018; Georges Des Aulnois et al., 2019; Osburn et al., 2023).

The detection of these potentially cyanotoxic populations in polyhaline waters is particularly important as they are potential vectors of cyanotoxics in APES regions that are far from conspicuous freshwater blooms. Reports of cyanotoxics in multiple trophic levels of marine coastal food webs are increasingly common (Brand et al., 2010; Ferrão-Filho and Kozlowsky-Suzuki, 2011; Bukaveckas et al., 2017; Preece et al., 2017; Anderson et al., 2023) as is our need to resolve toxin entry into the food web along the freshwater to marine continuum (Paerl et al., 2018b). Our results point to a more in-depth investigation of cosmopolitan genera as cyanotoxic vectors in the APES. Future studies should focus on impacts of potentially cyanotoxic populations on water quality and their respective competitive interactions within ecosystems. Furthermore, improved cultivation efforts, genomic sequencing, and *in situ* sampling hold promise for resolving the metabolisms and capabilities of these newly recognized populations of concern (Chen et al., 2021; Cabello-Yeves et al., 2022b).

## Conclusion

Our results—sequence data as well as abundance and biomass estimates—emphasize the following: (1) our knowledge of estuarine cyanobacterial diversity as well as their ecologies and ecosystem impact is currently limited, including relatively abundant populations; (2) picocyanobacteria are important contributors to phytoplankton biomass in the APES, especially understudied PC-Syn cells; and (3) several poorly studied cyanobacteria in the APES are potential cyanotoxic producers unexpectedly persisting or surviving in polyhaline waters. Our findings highlight important knowledge gaps that require future research. Specifically, we need to clarify metabolic pathways that can help explain the dominance of specific estuarine picocyanobacteria over less abundant yet phylogenetically related taxa, and determine the cyanotoxic production potential among genera with limited characterization within the APES (*Synechocystis*, *Planktothrix, Plectonema, Dolichospermum,* and others) to resolve the entry of cyanotoxics into local food webs. Furthermore, we should address primary and secondary metabolism in understudied and novel lineages, as some of these may impact water quality and biogeochemical cycling, and could also be beneficial for biotechnological advancements (e.g., diverse cyanobacteria in the AST). Overall, our results enhance the understanding of cyanobacteria in a large temperate estuarine system of biogeochemical and economic importance, particularly knowledge of key contributors to productivity, toxin production, and microbiome homeostasis amid environmental changes.

## Data Availability

Raw sequence data have been deposited in NCBI Genbank under Bioproject PRJNA1125422, Accessions: SRR29455238 - SRR29455322, https://www.ncbi.nlm.nih.gov/bioproject/PRJNA1125422.

## Footnotes

^1^ https://paerllab.web.unc.edu/modmon/
