# Supplementary material for: New perspectives on picocyanobacteria and understudied cyanobacterial diversity in the Albemarle Pamlico sound system, North Carolina, USA
Source: Front Microbiol. 2025 May 9;16:1539050. doi: 10.3389/fmicb.2025.1539050 (PMC12099654; doi:10.3389/fmicb.2025.1539050)
Supplement: Supplementary file 2 [file Data_Sheet_1.DOCX]

Supplementary Material For:

Shedding new light on picocyanobacteria and understudied cyanobacterial diversity in the Albemarle Pamlico Sound System, North Carolina, USA

Joel Sánchez-Gallego^1,3^, Nathaniel P. Curtis^1^, Hans W. Paerl^2^ , Ryan W. Paerl^1*^

**^1^**Department of Marine Earth and Atmospheric Sciences, North Carolina State University, Raleigh, North Carolina 27695-8208, United States of America

**^2^**Institute of Marine Sciences, Department of Earth, Marine and Environmental Sciences, University of North Carolina at Chapel Hill, Morehead City, NC 28557, United States of America

^3^Coiba Scientific Station, City of Knowledge, Calle Gustavo Lara, Bld. 145B, Clayton, 0843-01853, Panamá.


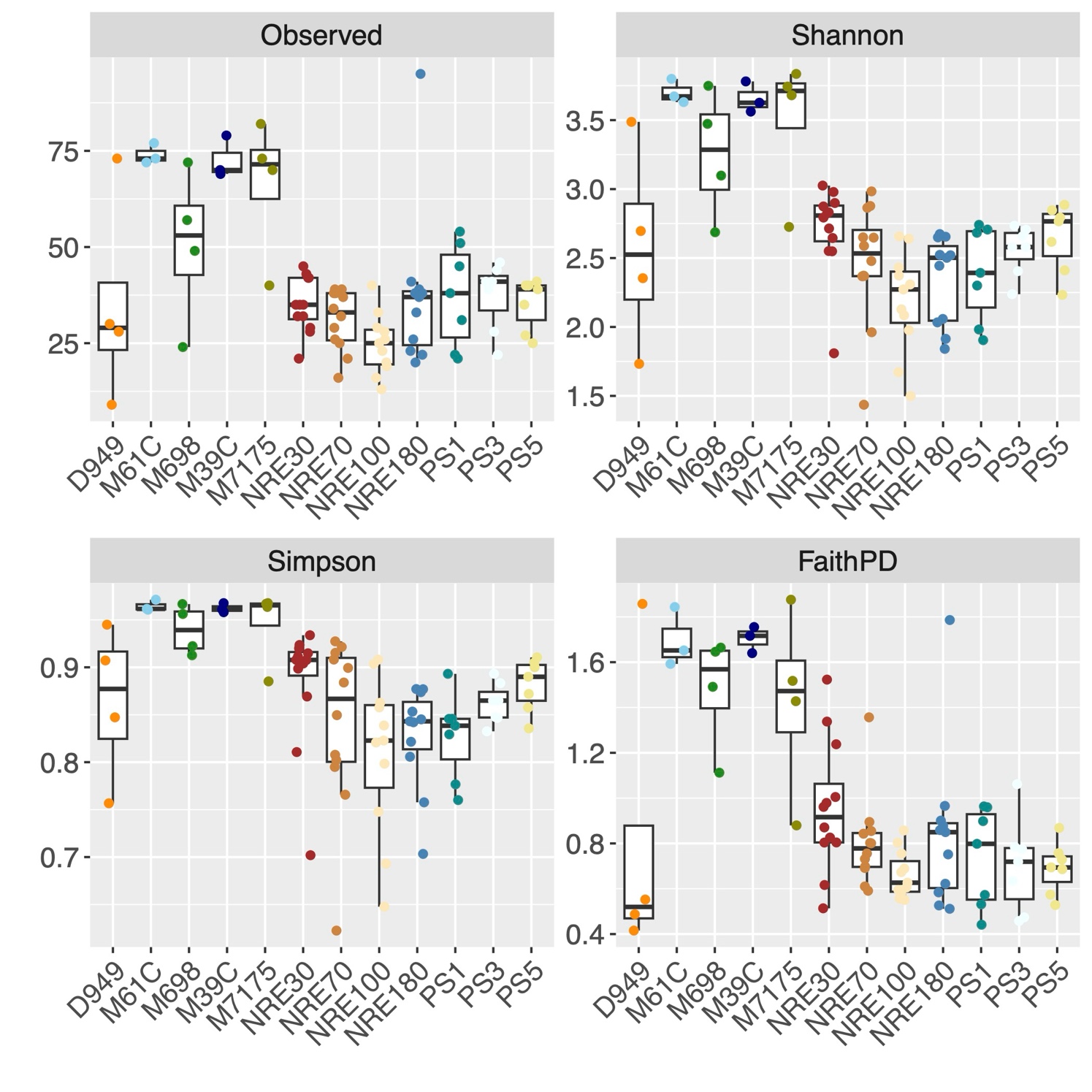


**Supplementary Figure S1.** Alpha diversity indexes at ASV level for cyanobacteria at individually sampled APES stations.

**Supplementary Figure S2.** Phylogeny of non-*Synechococcales* sequences from APES. A total of 181 ASVs and 123 reference sequences were incorporated into the tree to elucidate phylogenetic relationships. References were included based on the top BLASTn hits.


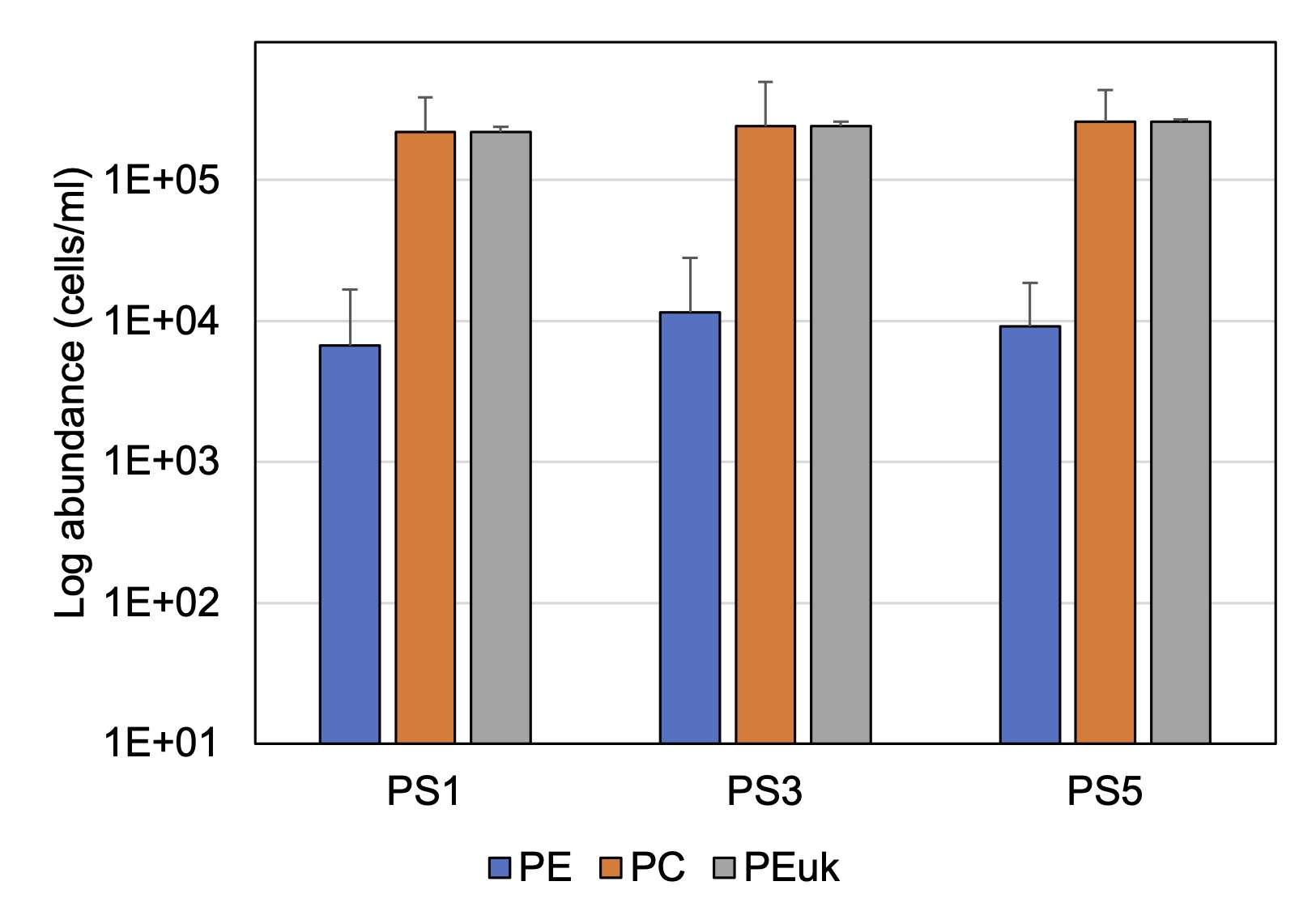


**Supplementary Figure S3.** Average abundance of picocyanobacterial morphotypes in PS samples. Means ± SD are shown.


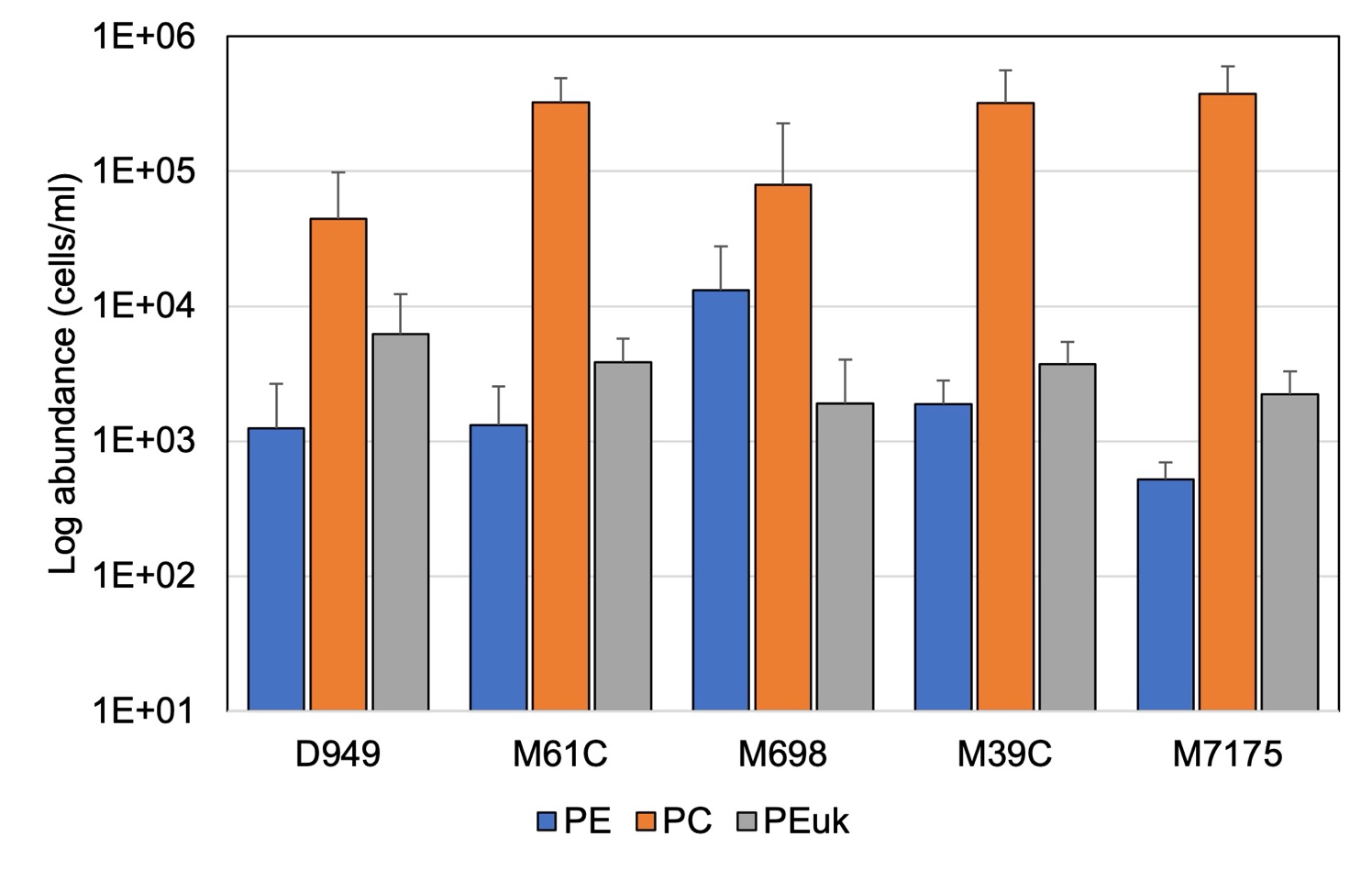


**Supplementary Figure S4.** Average abundance of picocyanobacterial morphotypes in AST samples. Means ± SD are shown.


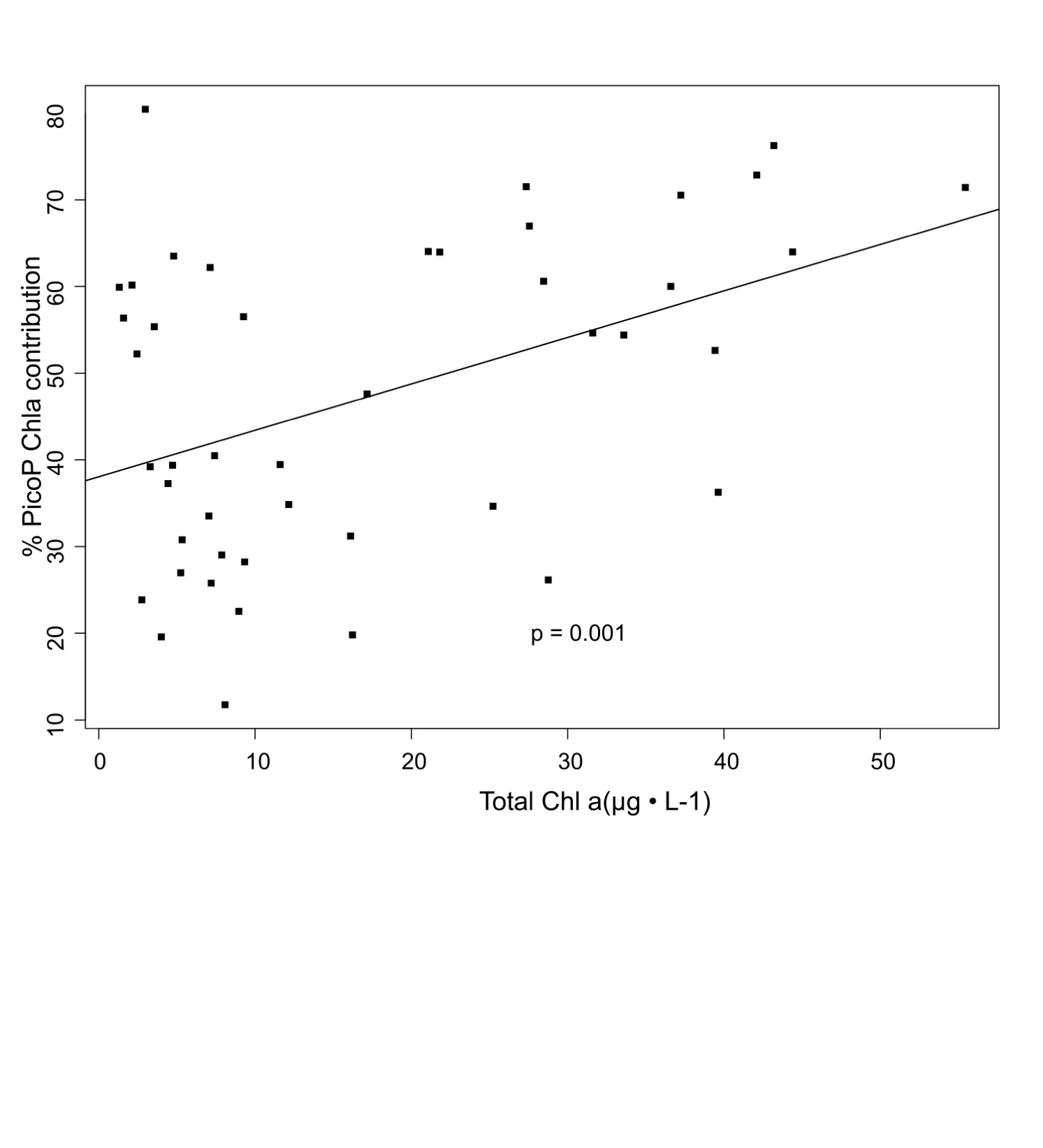


**Supplementary Figure S5.** PicoP Chl *a* contribution versus total Chl *a* concentration from five AST stations.


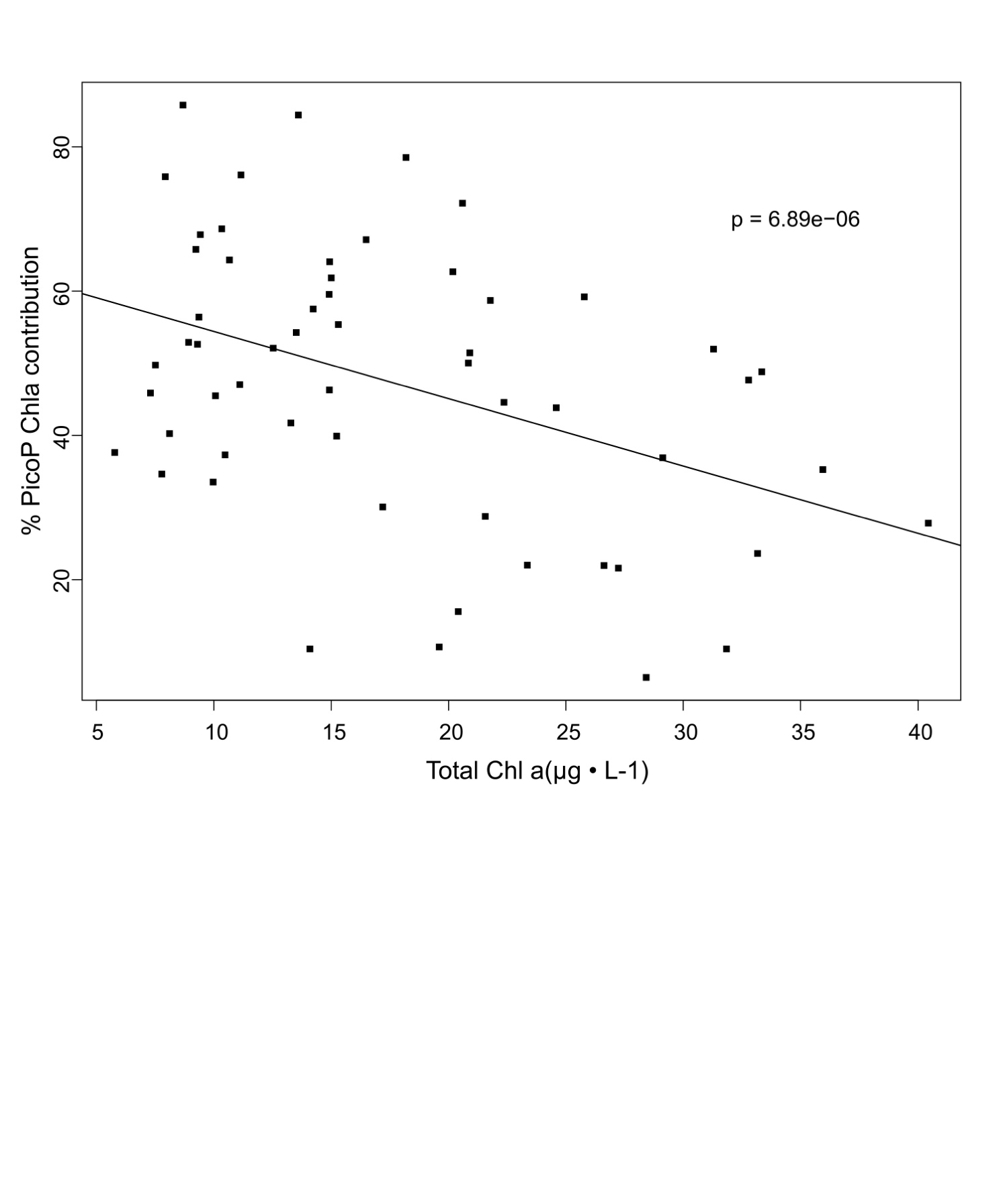


**Supplementary Figure S6.** PicoP Chl *a* contribution versus total Chl *a* concentration from three PS stations.

**Supplementary Figure S7A.** PicoP Chl *a* concentration versus total Chl *a* concentration from five AST stations across western to eastern Albemarle Sound. **Supplementary** **Figure S7B**. PicoP Chl *a* concentration versus total Chl *a* concentration from three Pamlico Sound (PS) stations.


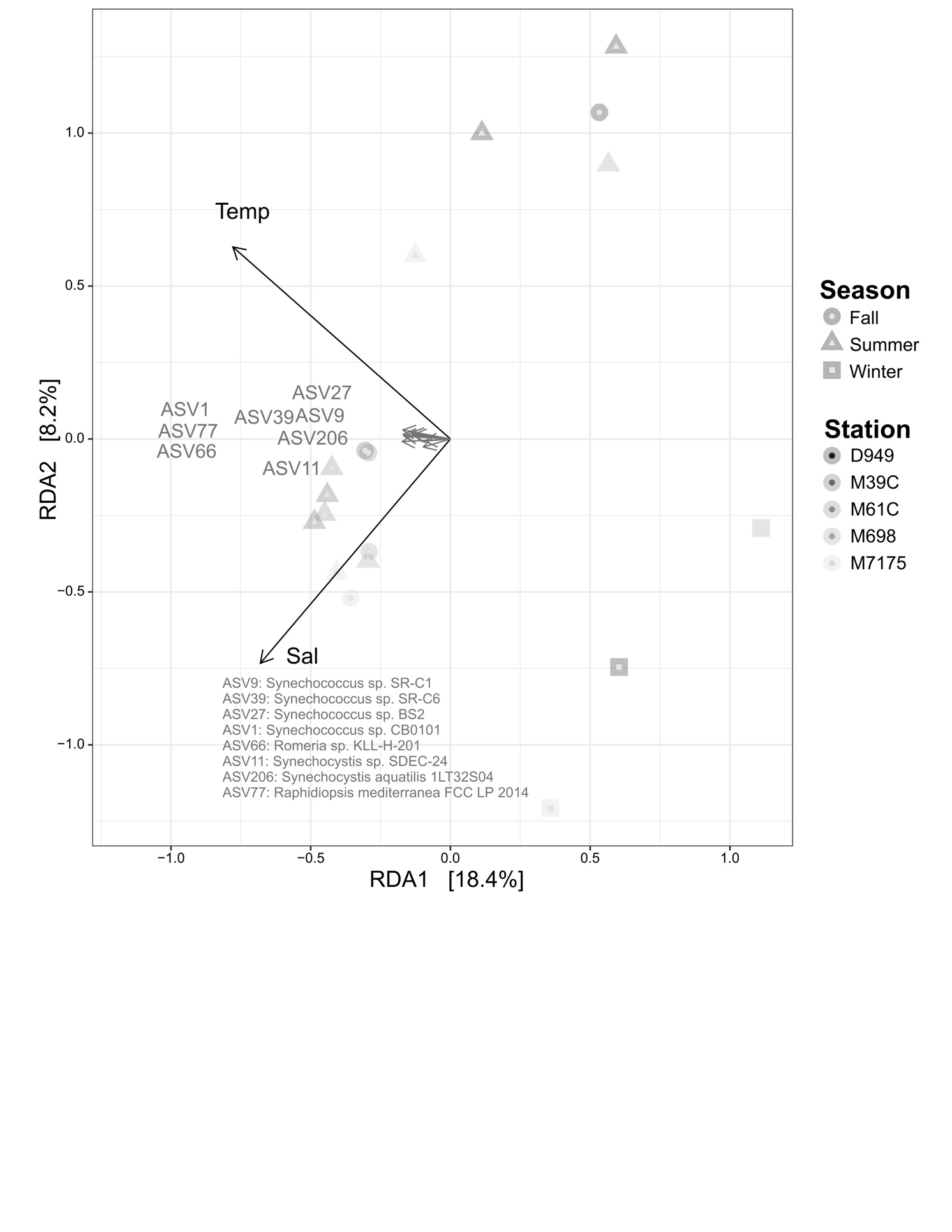


**Supplementary Figure S8.** Redundancy analysis ordination plot for AST cyanobacterial community composition in relation to environmental parameters. Only significant explanatory environmental factors (p ≤ 0.001) (black arrows) and ASVs (grey arrows) with goodness-of-fit ≥ 0.62 (grey) are included in the figure.


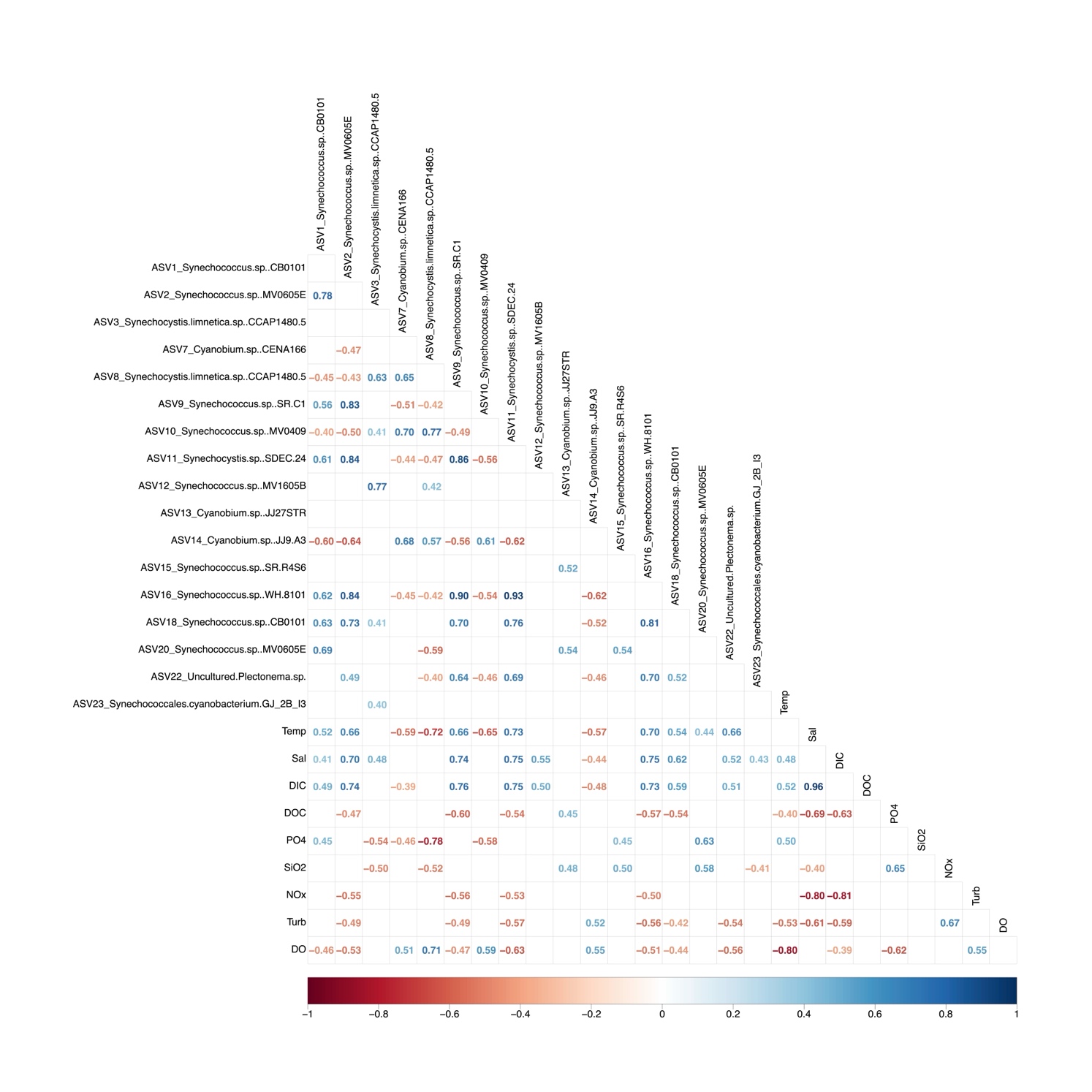


**Supplementary Figure S9.** Correlogram representing correlations (Spearman's rank correlation) between significant environmental parameters determined by the RDA and top 17 ASVs in NRE-PS. Insignificant correlations (p values ≥ 0.001) are represented by blanks spaces.


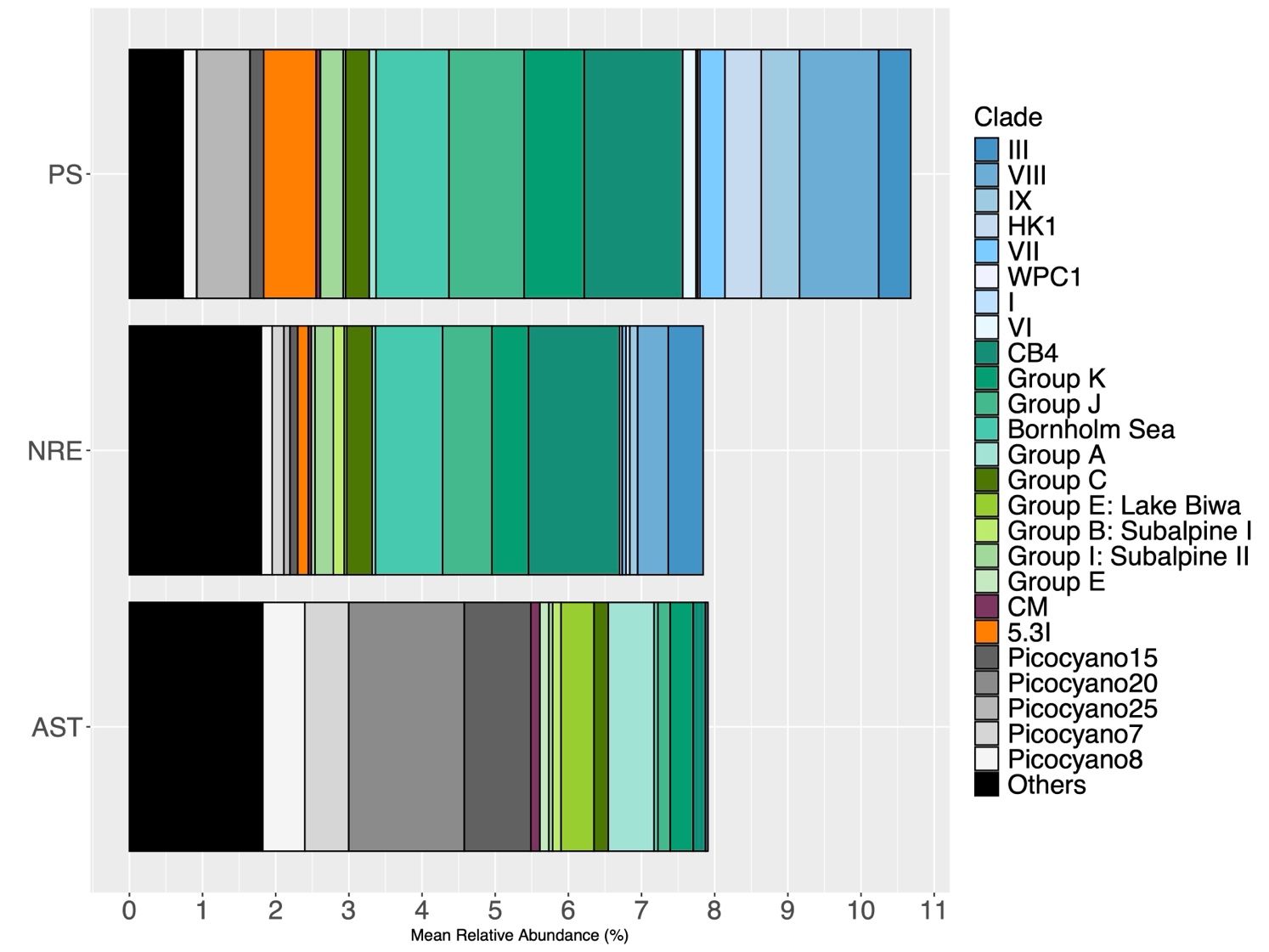


**Supplementary Figure S10**. *Synechococcales's* clade distribution across the three regions of the APES. Groups of *Synechococcus* subclusters (SC) are shown in green palette (SC 5.2), blue palette (SC 5.1), orange (5.3), purple palette (representing *Coelosphaeriaceae* & *Merismopediaceae*: *Synechocystis*, *Merismopedia*, *Snowella*, *Coleomoron, Woronichinia*), grey palette, (potential novel clades, “Picocyano”) and others in black (other 21 picocyanos’s clades).


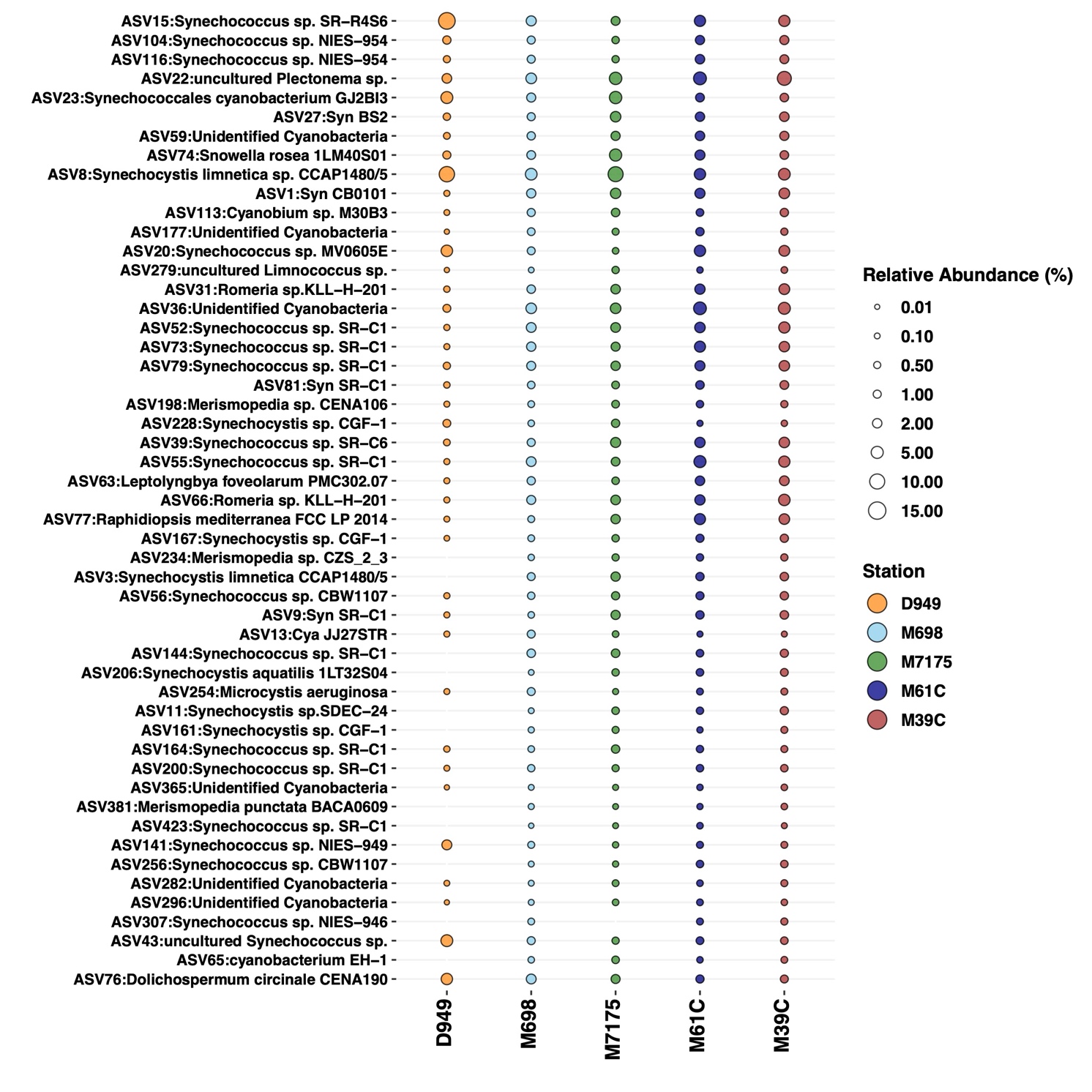


**Supplementary Figure S11.** A relative abundance bubble plot representing the core/prevalent ASVs at only AST stations. Samples were grouped by stations and each bubble represents the mean of each ASV by station. ASVs were ordered based on prevalence, with the most prevalent at the top and the least prevalent at the bottom.


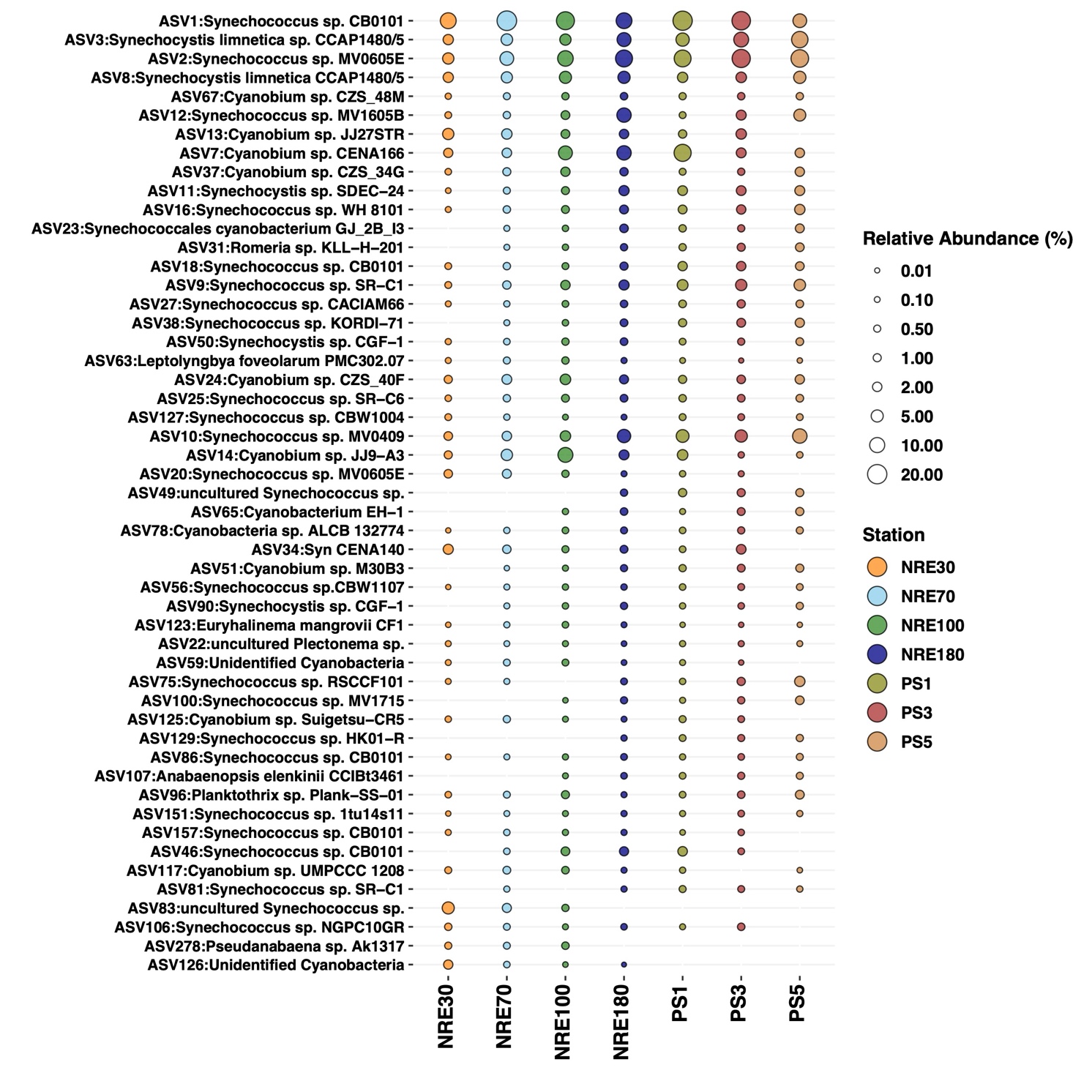


**Supplementary Figure S12.** An abundance bubble plot representing the core/prevalent ASVs across only NRE-PS stations. Each bubble represents the mean of each ASV by station. ASVs were ordered based on prevalence, with the most prevalent at the top and the least prevalent at the bottom.


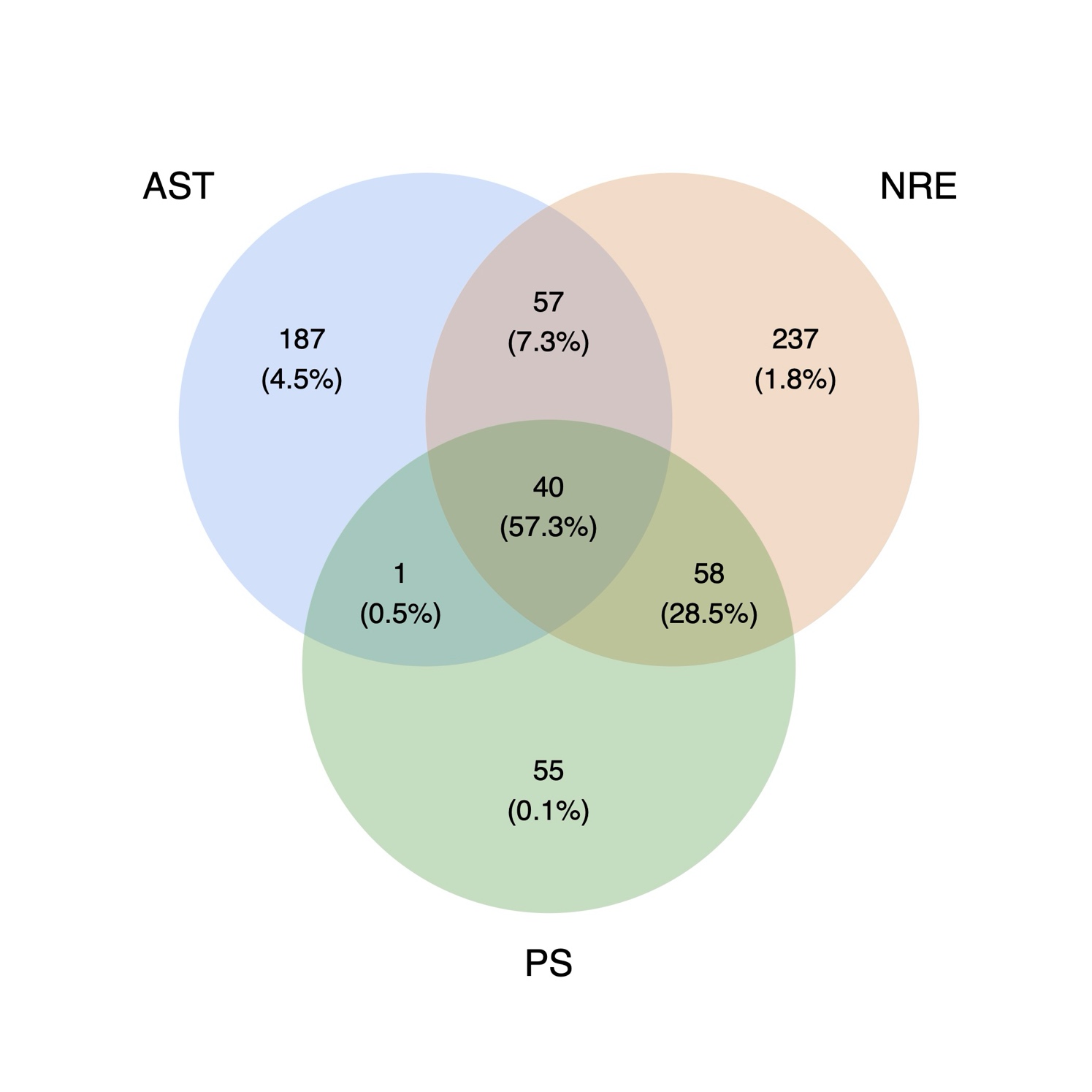


**Supplementary Figure S13**. A Venn diagram illustrating distinct and overlapping cyanobacterial ASVs occurring in APES regions. The shared cyanobacterial core ASVs occur in the middle overlapping region. The integers are the ASV numbers in each region. The percentage data is the sequence number/total sequence number.
